# Dectin-2 is a primary receptor for NLRP3 inflammasome activation in dendritic cell response to *Histoplasma capsulatum*

**DOI:** 10.1371/journal.ppat.1006485

**Published:** 2017-07-03

**Authors:** Tzu-Hsuan Chang, Juin-Hua Huang, Hsiu-Chao Lin, Wen-Yu Chen, Yu-Hsiang Lee, Li-Chung Hsu, Mihai G. Netea, Jenny P.-Y. Ting, Betty A. Wu-Hsieh

**Affiliations:** 1 Graduate Institute of Immunology, National Taiwan University College of Medicine, Taipei, Taiwan; 2 Graduate Institute of Molecular Medicine, National Taiwan University College of Medicine, Taipei, Taiwan; 3 Department of Internal Medicine and Radboud Center for Infectious Diseases, Radboud University Medical Center, Nijmegen, The Netherlands; 4 Department of Microbiology and Immunology, University of North Carolina, Chapel Hill, North Carolina, United States of America; 5 Lineberger Comprehensive Cancer Center, University of North Carolina, Chapel Hill, North Carolina, United States of America; University of California, San Francisco, UNITED STATES

## Abstract

Inflammasome is an intracellular protein complex that serves as cytosolic pattern recognition receptor (PRR) to engage with pathogens and to process cytokines of the interleukin-1 (IL-1) family into bioactive molecules. It has been established that interleukin-1β (IL-1β) is important to host defense against *Histoplasma capsulatum* infection. However, the detailed mechanism of how *H*. *capsulatum* induces inflammasome activation leading to IL-1β production has not been studied. Here, we showed in dendritic cells (DCs) that *H*. *capsulatum* triggers caspase-1 activation and IL-1β production through NLRP3 inflammasome. By reciprocal blocking of Dectin-1 or Dectin-2 in single receptor-deficient DCs and cells from *Clec4n*^*-/-*^, *Clec7a*^*-/-*^, and *Clec7a*^*-/-*^*Clec4n*^*-/-*^ mice, we discovered that while Dectin-2 operates as a primary receptor, Dectin-1 serves as a secondary one for NLRP3 inflammasome. In addition, both receptors trigger Syk-JNK signal pathway to activate signal 1 (pro-IL-1β synthesis) and signal 2 (activation of caspase-1). Results of pulmonary infection with *H*. *capsulatum* showed that CD103^+^ DCs are one of the major producers of IL-1β and Dectin-2 and Dectin-1 double deficiency abolishes their IL-1β response to the fungus. While K^+^ efflux and cathepsin B (but not ROS) function as signal 2, viable but not heat-killed *H*. *capsulatum* triggers profound lysosomal rupture leading to cathepsin B release. Interestingly, cathepsin B release is regulated by ERK/JNK downstream of Dectin-2 and Dectin-1. Our study demonstrates for the first time the unique roles of Dectin-2 and Dectin-1 in triggering Syk-JNK to activate signal 1 and 2 for *H*. *capsulatum*-induced NLRP3 inflammasome activation.

## Author summary

*Histoplasma capsulatum* is a dimorphic fungal pathogen. The microconidia and hyphal elements are breathed in and transform to become yeasts in the lungs. Histoplasmosis occurs worldwide and endemic in mid-western United States. The infection is primarily in the lungs that can become disseminated and cause fatal disease when left untreated. It was reported that IL-1β is important to host defense against *H*. *capsulatum* infection, but the detailed mechanism of how myeloid cells respond to this fungal pathogen and which receptor(s) is involved to induce IL-1β production is largely unknown. We demonstrate in this study that *H*. *capsulatum*-induced caspase-1 activation leading to IL-1β production is NLRP3-dependent. NLRP3-deficienct mice succumb to otherwise sublethal *H*. *capsulatum* infection. Although the role of Dectin-1 in fungus-induced NLRP3 inflammasome is well-established, we found that Dectin-2 serves as a primary receptor and Dectin-1 plays a secondary role in inducing Syk-JNK signaling to mediate NLRP3 inflammasome in response to *H*. *capsulatum*. In addition, while both K^+^ efflux and cathepsin B function as signal 2, the viability *H*. *capsulatum* affects the amounts of cathepsin B release. Our study is the first to reveal the roles of Dectin-2 and Dectin-1 and the downstream signaling events in fungal pathogen-induced NLRP3 inflammasome.

## Introduction

Inflammasome is a large intracellular multimeric protein platform which is activated upon infection or stress [1]. The function of inflammasome is to drive the maturation of proinflammatory cytokines of the IL-1 family, most importantly IL-1β and IL-18 and induction of inflammatory cell death [2]. Among all identified inflammasome complexes, NLRP3 inflammasome is well-characterized. It is generally accepted that NLRP3-driven processing and secretion of IL-1β and IL-18 in macrophage and DC require two signals [3]. Signal 1 is induced by engagement of pathogen-associated molecular patterns (PAMPs) with pattern recognition receptors (PRRs) leading to gene transcription and synthesis of NLRP3, inactive pro-IL-1β and pro-IL-18 [4]. Signal 2 induces the assembly of inflammasome complex and activates caspase-1 to facilitate pro-IL-1β and pro-IL-18 cleavage into their mature forms, and is induced by intracellular events including reactive oxygen species (ROS) production, potassium (K^+^) efflux, cathepsin B release, calcium influx and mitochondrial destabilization [5–9]. There are multiple PAMPs on a single fungal pathogen. It is of interest to determine the complex interaction between a fungus and the host cell and how the interaction triggers either signal 1 or 2 or both for inflammasome activation.

*Histoplasma capsulatum* is a dimorphic fungal pathogen. The microconidia and mycelial fragments of *H*. *capsulatum* spread in the air and infect humans through inhalation [10, 11]. *H*. *capsulatum* stimulates mouse dendritic cell (DC) to secrete pro-inflammatory cytokines such as IL-1β, IL-18, TNF and IL-6 [12]. Human DC phagocytoses *H*. *capsulatum* yeasts through fibronectin receptor VLA-5 and kills the organism via phagolysosomal fusion [13, 14]. A recent study showed that CD103^+^ conventional DC in the lungs produces IFN-I to restrict the growth of *H*. *capsulatum* during pulmonary infection [15]. These studies point to a crucial role of DC in secreting cytokines and killing *H*. *capsulatum* during early phase of infection [13–15]. There is still much to be learned about the detailed mechanisms of cytokine production by DC through interaction with *H*. *capsulatum*.

The fungal pathogens *Candida albicans*, *Aspergillus fumigatus*, *Cryptococcus neoformans*, *Microsporum canis* and *Malassezia* spp. induce inflammasome activation [16–21]. In a systemic *C*. *albicans* infection model, NLRP3 or caspase-1 deficiency leads to increased fungal burdens and higher mortality [16]. In protection against mucosal candidiasis, NLRC4 functions at the level of mucosal stroma and NLRP3 at both the hematopoietic and stromal compartments [21]. AIM2 and NLRP3 are both required for mice that are treated with immunosuppressive agents to confine *A*. *fumigatus* in inflammatory foci after intranasal inoculation with conidia [18]. In pulmonary infection with acapsular form of *C*. *neoformans*, NLRP3 inflammasome activation results in immune cell infiltration and effective fungal clearance [17]. While elevated IL-1β in the lungs of mice infected with *H*. *capsulatum* plays a critical role in host defense against *H*. *capsulatum* [22], it has never been determined which mechanisms are involved in inflammasome activation and IL-1β production.

Hyphal forms of *C*. *albicans* recognized by either TLR-2 or Dectin-1 on bone marrow-derived macrophage triggers the synthesis of pro-IL-1β through Syk kinase [23]. In addition to triggering pro-IL-1β accumulation, Syk signaling is also involved in ROS production and caspase-1 activity in bone marrow-derived dendritic cells (BMDCs) stimulated by *C*. *albicans* [16]. Interestingly, in contrast to stimulation by hyphal forms, stimulation of BMDC by yeast form of *C*. *albicans* for IL-1β production is mediated by Dectin-2 through MAPKs signaling [24, 25]. Both CR3 and Dectin-1 are involved in macrophage TNF and IL-6 response to *H*. *capsulatum* through activation of Syk-JNK-AP-1 pathway [26]. In studying vaccine immunity, Wang *et al*. reported that Dectin-1 and Dectin-2 fusion proteins separately bind to *H*. *capsulatum* and that CARD9 signaling is important for development of Th17 cells and adaptive immunity against *H*. *capsulatum* [27].

In this study, we demonstrated that *H*. *capsulatum* induced NLRP3 inflammasome for IL-1β production in BMDCs. Dectin-2 was the primary receptor that mediated both signal 1 and 2 for NLRP3 inflammasome. Results of reciprocal blocking of Dectin-1 or Dectin-2 in Dectin-2- and Dectin-1-deficient cells and that of using single- and double-deficient cells showed that Dectin-1 played a role as a secondary receptor. Both Dectin-2 and Dectin-1 activated the Syk/JNK signaling pathway but the role of Dectin-1 was less prominent than Dectin-2. Pulmonary infection results showed that CD103^+^ DCs are one of the major sources of IL-1β, and Dectin-2 and Dectin-1 together mediated the IL-1β response of CD103^+^ DC to *H*. *capsulatum* infection. Both K^+^ efflux and cathepsin B release but not ROS functioned as signal 2 for *H*. *capsulatum*-induced NLRP3 inflammasome. While Dectin-2 did not affect K^+^ efflux, signals from Dectin-1 and Dectin-2 cooperatively regulated cathepsin B release. Our work revealed the roles of Dectin-2 and Dectin-1 in inducing signals for activation of NLRP3 inflammasome during fungal infection.

## Results

### *H*. *capsulatum*-induced IL-1β production in dendritic cells is caspase-1- and NLRP3-dependent

**Fig 1:**
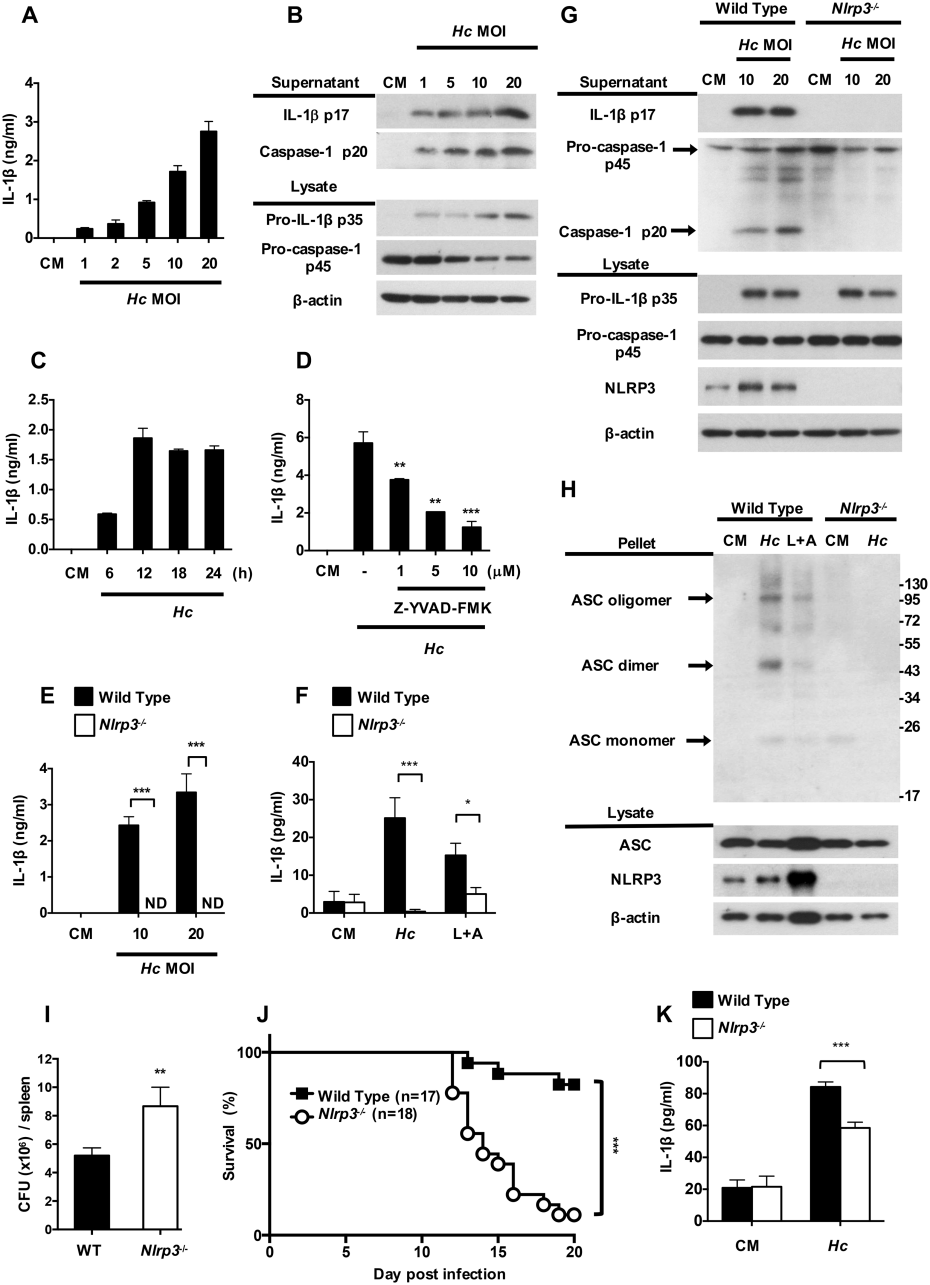
*H*. *capsulatum* induces NLRP3-dependent inflammasome activation. (A, B and C) BMDCs from wild type mice were stimulated with live *H*. *capsulatum* at (A and B) different MOI and (C) at MOI of 20 for 6, 12, 18 and 24 h. (D) BMDCs were pretreated with caspase-1 inhibitor (Z-YVAD-FMK) at different concentrations before stimulation with *H*. *capsulatum* for another 18 h. (E, G and H) BMDCs and (F) sorted MHCII^+^CD11c^+^ splenic DCs from wild type and NLRP3-deficient (*Nlrp3*^*-/-*^) mice were stimulated with *H*. *capsulatum* at MOI of 10 and 20 for 18 h. (B and G) Cell-free supernatants and cell lysates were analyzed by Western blotting with indicated antibodies. (H) ASC pyroptosome and inflammasome components were analyzed in cell pellets and cell lysates, respectively. Stimulation with LPS (500 ng/ml, 6 h) plus ATP (5 mM, 30 minutes) (L+A) was used as a positive control for NLRP3-dependent IL-1β induction. (I and J) Wild type and NLRP3-deficient mice were intravenously infected with *H*. *capsulatum* (1 × 10^7^). (I) Fungal burden in the spleen was determined on day 11 after infection. It is shown as CFU per organ (n = 6). (J) Survival was analyzed by log-rank test. (K) Wild type and NLRP3-deficient mice were intravenously infected with 2.5 × 10^5^ of *H*. *capsulatum*. MHCII^+^CD11c^+^ splenic DCs were sorted from mice on day 5 after infection and stimulated with live *H*. *capsulatum* at MOI of 1 for 18 h. (A, C, D, E, F and K) IL-1β in cell-free supernatants were quantified by ELISA (n = 3). One representative of three (A, B, C, D and E) or two (F, G, H, I and J) independent experiments is presented. ND, not detectable. CM, complete medium. * *p* < 0.05, ** *p* < 0.01, *** *p* < 0.001 [one-way ANOVA with Tuckey post-hoc analysis (A, B and D); 2-tailed *t*-test (E, F, I and K); log-rank test (J)].

Inflammasome activation is comprised of two signals: the first involves an increase in the transcription and expression of certain inflammasome components, and the second is comprised of the assembly of inflammasome protein components resulting in pro-caspase-1 activation to caspase-1. Inflammasome is reported to be associated with and acts as a defense mechanism against fungal infections [28]. Here, we sought to investigate whether *H*. *capsulatum* triggers inflammasome activation in DC. BMDCs were stimulated with viable *H*. *capsulatum* yeast cells at different yeast-to-cell (MOI) ratios. IL-1β and caspase-1 p20 were detected in culture supernatants at a MOI as low as 1 and their levels increased with increasing MOI (Fig 1A and 1B). IL-1β production was detectable at as early as 6 h, peaked at 12 h and maintained at the peak level until 24 h after stimulation (Fig 1C). Treatment with caspase-1 inhibitor (Z-YVAD-FMK) significantly reduced IL-1β production in a dose-dependent manner (Fig 1D). NLRP3 deficiency completely abolished IL-1β production in both BMDCs and MHCII^+^CD11c^+^ splenic DCs while it did not affect the production of TNF (Fig 1E and 1F and S1 Fig). Western blotting analysis showed that *H*. *capsulatum* caused increased expression of pro-IL-1β and NLRP3. While the absence of NLRP3 did not affect pro-IL-1β and pro-caspase-1 p45, the secretion of IL-1β p17 and caspase-1 p20 was completely abrogated in *Nlrp3*^-/-^ cells (Fig 1G). A protein-crosslinking experiment showed that *H*. *capsulatum* induced robust NLRP3-dependent ASC oligomerization, which reflected inflammasome assembly (Fig 1H). *Nlrp3*^-/-^ mice had greater fungal burden (Fig 1I) and poorer survival (Fig 1J) compared to wild type mice after intravenous infection with *H*. *capsulatum*. It appears that higher fungal burden caused by NLRP3 deficiency may be one of the contributing factors to poor survival. In addition, splenic DCs from *Nlrp3*^-/-^ infected-mice produced lower IL-1β compared with cells from infected wild type mice (Fig 1K). These results together show that *H*. *capsulatum* induces IL-1β response through activation of both signal 1 and signal 2 of an NLRP3-dependent inflammasome in DCs and that NLRP3 is important to protection against histoplasmosis.

### Dectin-2 mediates inflammasome activation in *H*. *capsulatum*-stimulated BMDCs

**Fig 2:**
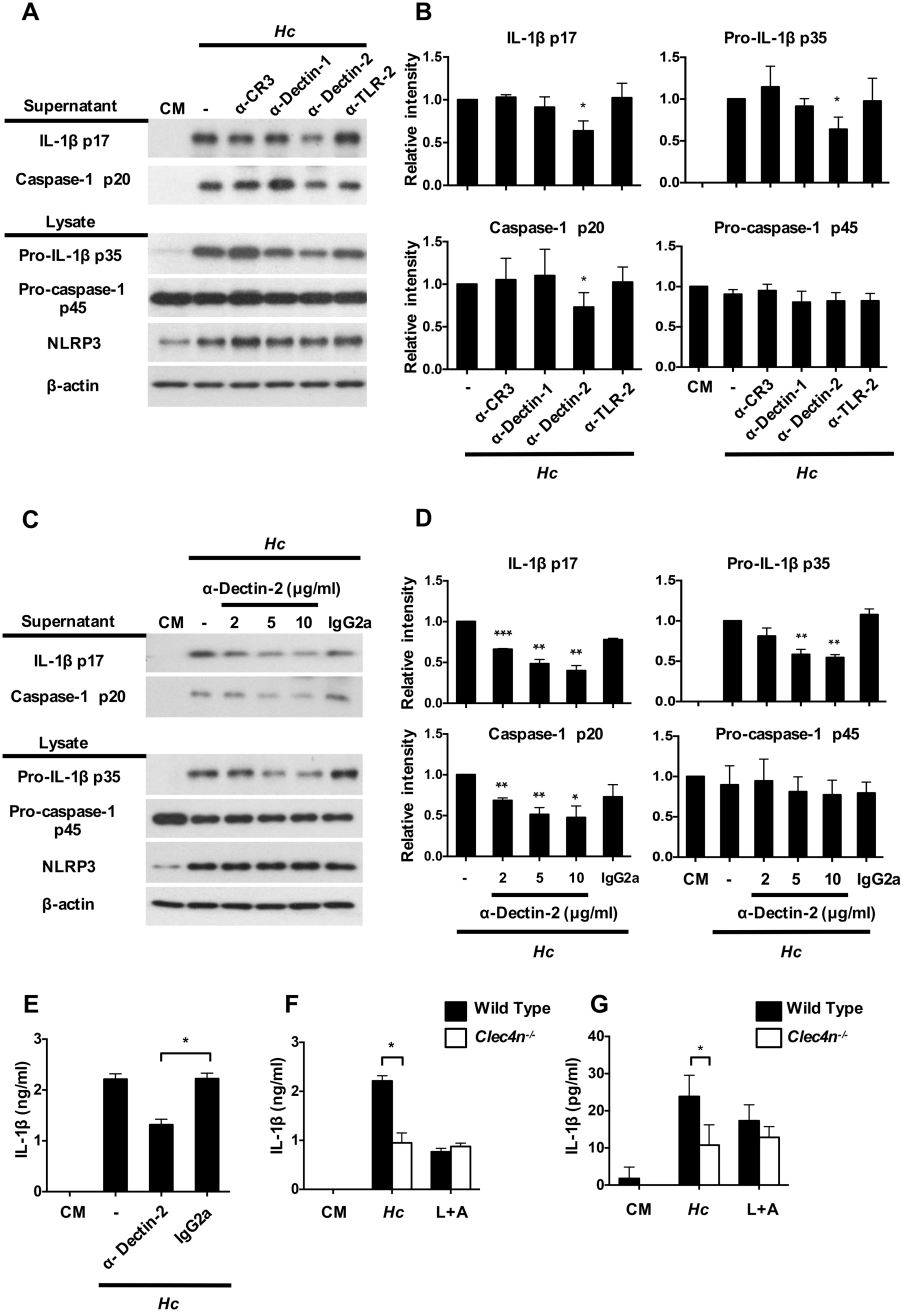
Dectin-2 mediates inflammasome activation upon *H*. *capsulatum* stimulation. (A) BMDCs were pretreated with anti-CR3, -Dectin-1, -Dectin-2 and -TLR-2 (2 μg/ml) and (C) different concentrations of anti-Dectin-2 blocking antibodies at 2, 5, and 10 μg/ml for 1 h before stimulation with *H*. *capsulatum* for another 18 h. IgG2a was used as an isotype control. Cell-free supernatants and cell lysates were analyzed by Western blotting with indicated antibodies. (B and D) Relative intensity of secreted IL-1β p17, caspase-1 p20, pro-IL-1β p35 and pro-caspase-1 p45 were quantified by ImageJ (n = 3). Data were pooled from three independent experiments. (E and F) BMDCs and (G) sorted-MHCII^+^CD11c^+^ from the spleens of wild type and Dectin-2-deficient (*Clec4n*^-/-^) mice were treated with or without blocking antibody against Dectin-2 before stimulation with *H*. *capsulatum*. Stimulation by LPS plus ATP (L+A) was used as a positive control for IL-1β induction. Cell-free supernatants were collected at 18 h after stimulation. Secreted IL-1β was quantified by ELISA (n = 3). Error bars indicate standard deviation of the mean. One representative of three independent experiments is presented. * *p* < 0.05, ** *p* < 0.01, *** *p* < 0.001 [one-way ANOVA with Tuckey post-hoc analysis (B and D); 2-tailed *t*-test (E, F and G)].

We then used blocking antibodies to explore surface receptor(s) that is involved in inflammasome activation by *H*. *capsulatum*. Results show that BMDCs treated with anti-Dectin-2, but not anti-CR3, -Dectin-1 or -TLR-2 blocking antibody significantly reduced *H*. *capsulatum*-induced pro-IL-1β p35, IL-1β p17 and caspase-1 p20 expressions (Fig 2A and 2B) and Dectin-2 blocking antibody dose-dependently reduced pro-IL-1β p35, IL-1β p17 and caspase-1 p20 production (Fig 2C and 2D). Thus, it appears that Dectin-2 is involved in pro-IL-1β synthesis, IL-1β maturation and caspase-1 activation in response to *H*. *capsulatum*. Results in Fig 2E and 2F show that Dectin-2 deficiency (*Clec4n*^-/-^ cells) exhibited reduction of IL-1β secretion, which is comparable with wild type BMDCs treated with anti-Dectin-2 antibody. Sorted MHCII^+^CD11c^+^ splenic DCs from *Clec4n*^-/-^ mice also produced lower IL-1β compared with their wild type counterparts (Fig 2G). Since Dectin-2 signal is transduced through Fc receptor γ chain (FcRγ), we studied inflammasome activation in *FcRγ*^-/-^ BMDCs. We found that FcRγ deficiency, like Dectin-2 deficiency, significantly reduced IL-1β and caspase-1 p20 (S2 Fig). These results together indicate that through interaction with Dectin-2, *H*. *capsulatum* triggers both signal 1 and signal 2 for inflammasome activation in BMDC, demonstrating the importance of Dectin-2 in *H*. *capsulatum*-induced inflammasome activation in DC.

### Syk and its downstream ERK and JNK regulate inflammasome activation after stimulation by *H*. *capsulatum*

**Fig 3:**
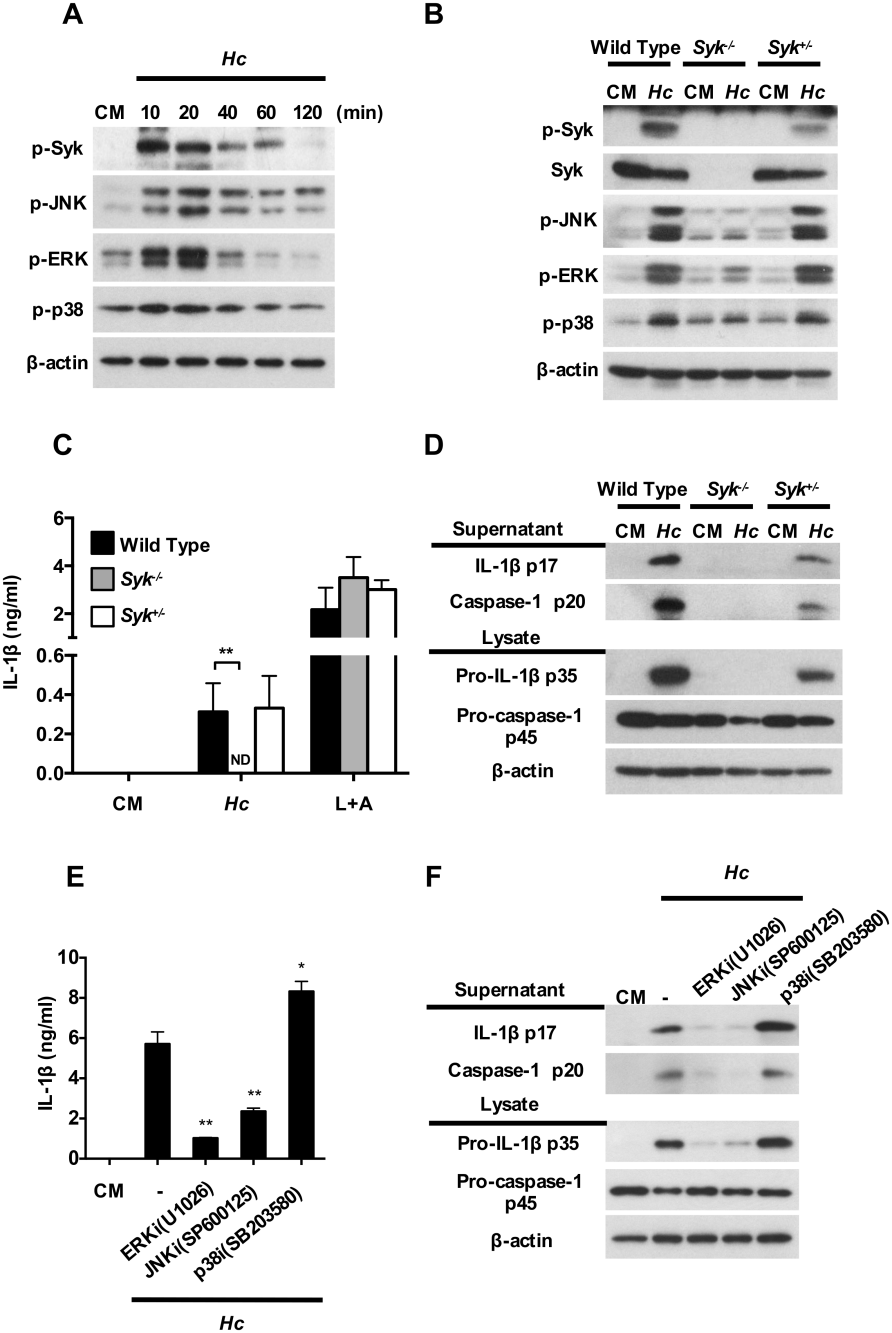
Syk-ERK/JNK pathway regulates inflammasome activation in response to *H*. *capsulatum*. (A) Wild type BMDCs were stimulated with or without (0 min) *H*. *capsulatum* for 10, 20, 40, 60, and 120 min. Cell lysates were subjected to Western blotting for detection of phosphorylated Syk, JNK, ERK and p38. (B, C and D) Fetal liver-derived dendritic cells from wild type, Syk-deficient and Syk heterozygous fetuses were stimulated with *H*. *capsulatum* for (B) 20 minutes and (C and D) 18 h. Stimulation by LPS plus ATP (L+A) was used as a positive control for IL-1β induction (E and F) BMDCs were pretreated with ERK inhibitor (U1026), JNK inhibitor (SP600125) and p38 inhibitor (SB203580) at 10 μM for 1 h before stimulation with *H*. *capsulatum*. (C and E) Culture supernatants were collected and quantified for IL-1β by ELISA (n = 3–5). (B, D and F) Cell-free supernatants and cell lysates were analyzed for inflammasome components by Western blotting. β-actin was used as an internal control. Error bars indicate standard deviation of the mean. One representative of three (A, C, D and E) or two (B and F) independent experiments is presented. * *p* < 0.05, ** *p* < 0.01 [2-tailed *t*-test (C); one-way ANOVA with Tuckey post-hoc analysis (E)].

It has been reported that Dectin-2 coupling to Syk leads to downstream activation of MAPKs in response to *C*. *albicans* [25]. Whether Syk-MAPK signaling pathway is involved in *H*. *capsulatum*-induced inflammasome activation is still unclear. We analyzed Syk, JNK, ERK and p38 phosphorylation in BMDCs after stimulation with *H*. *capsulatum*. Western blotting showed that phosphorylation of Syk, JNK, ERK and p38 occurred as early as 10 min after *H*. *capsulatum* stimulation (Fig 3A). The role of Syk in *H*. *capsulatum*-induced MAPKs and inflammasome activation was validated in Syk-deficient fetal liver-derived dendritic cells (FLDCs). Results showed that phosphorylation of JNK, ERK and p38 was largely diminished in Syk-deficient cells upon *H*. *capsulatum* stimulation (Fig 3B), which demonstrates that MAPKs activation is downstream of Syk. While Syk-deficiency completely abolished FLDCs IL-1β secretion upon *H*. *capsulatum* stimulation, it did not affect IL-1β response to LPS plus ATP stimulation showing the specificity of this pathway (Fig 3C). Western blotting analysis also showed that in the absence of Syk, pro-IL-1β expression was abolished, which is congruent with the absence of mature IL-1β p17 production and indicates that Syk was required for signal 1 priming (Fig 3D). In addition, pro-caspase-1 p45 was partially reduced but caspase-1 p20 was completely absent in Syk deficiency, suggesting that Syk is likely necessary for optimal signal 2 activation (Fig 3D and S3 Fig). Pharmacological inhibition of either ERK or JNK dramatically reduced the production of pro-IL-1β p35, IL-1β p17 and caspase-1 p20 upon *H*. *capsulatum* stimulation (Fig 3E and 3F). Interestingly, inhibition of p38 increased pro-IL-1β expression that led to increase IL-1β secretion (Fig 3E and 3F). Again, these inhibitors greatly diminished signal 1 by reducing pro-IL-1β production, but also affected signal 2 by reducing the conversion of pro-caspase-1 to active caspase-1. Together, these data demonstrate that *H*. *capsulatum*-induced inflammasome activation is through Syk and its downstream ERK and JNK activation.

### Dectin-1 deficiency further reduces inflammasome activation in the absence of functional Dectin-2 through Syk-JNK signal pathway

**Fig 4:**
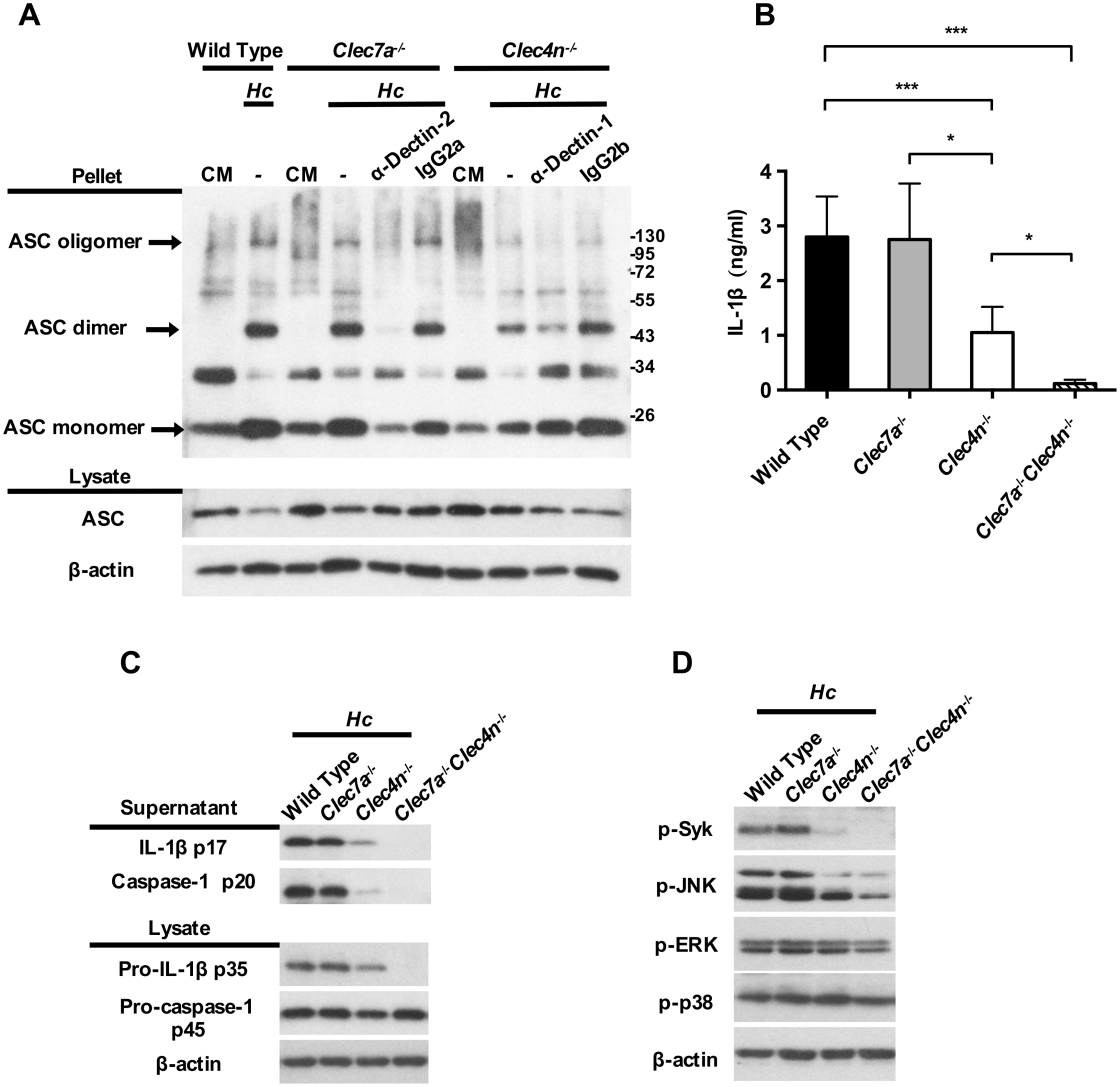
Dectin-1 deficiency further reduces inflammasome activation in the absence of functional Dectin-2 through Syk-JNK signal pathway. BMDCs from wild type, Dectin-2-deficient (*Clec4n*^-/-^), Dectin-1-deficient (*Clec7a*^-/-^), and Dectin-1 and Dectin-2 double-deficient (*Clec7a*^-/-^*Clec4n*^-/-^) mice were pretreated with (A) or without (B, C and D) anti-Dectin-1 or -Dectin-2 blocking antibody (2 μg/ml) for 1 h before stimulation with *H*. *capsulatum*. (A, C and D) Cell-free supernatants, cell lysates and cell pellets were subjected to Western blotting to analyze ASC oligomerization (A), inflammasome components (C) and signaling molecules (D). IgG2a and IgG2b were used as isotype controls. (B) Cell culture supernatants were collected at 18 h after stimulation. Secreted IL-1β was quantified by ELISA (n = 5). Error bars indicate standard deviation of the mean. One representative of two independent experiments is presented. * *p* < 0.05, ** *p* < 0.01, *** *p* < 0.001 [one-way ANOVA with Tukey post-hoc analysis (B)].

Our data in Figs 2 and 3 demonstrated that while Syk deficiency completely abolished IL-1β production, Dectin-2 deficiency reduced IL-1β production by only ~50%. It is reported that blocking Dectin-2 in Dectin-1-deficient BMDC leads to further reduction in TNF and IL-10 production in response to *C*. *albicans* compared to Dectin-1 deficiency alone [25]. Flow cytometric analysis results showed that Dectin-1 and Dectin-2 single deficiency did not affect the expression of the other receptor nor the uptake of *H*. *capsulatum* (S4 and S5 Figs). We used blocking antibodies and receptor single- and double-deficient BMDCs to study whether Dectin-1 works together with Dectin-2 to transduce downstream signaling for inflammasome activation. Western blotting analysis and ELISA results showed that blocking Dectin-1 and Dectin-2 in Dectin-2-deficient and Dectin-1-deficient, respectively, reduced not only the secretion of mature IL-1β p17 (S6A and S6B Fig), but also the synthesis of pro-IL-1β p35, the active form of caspase-1 p20, and ASC oligomerization, compared to wild type cells and cells with either receptor deficiency alone (S6B Fig and Fig 4A). Dectin-1 and Dectin-2 double deficiency completely abolished IL-1β production (Fig 4B and 4C) and pro-IL-1β p35, caspase-1 p20 (Fig 4C). It appears that, in the presence of Dectin-2, Dectin-1 does not function to respond to *H*. *capsulatum* for inflammasome activation. Yet in the absence of functional Dectin-2, recognition of *H*. *capsulatum* by Dectin-1 is involved in triggering both signal 1 and signal 2.

Interestingly, while Dectin-2 (but not Dectin-1) deficiency significantly reduced p-Syk and p-JNK, Dectin-1 and Dectin-2 double deficiency and blockade of Dectin-2 in Dectin-1-deficient cells further diminished Syk and JNK phosphorylation (Fig 4D and S7, S8A and S8B Figs). Blockade of Dectin-1 in Dectin-2-deficient cells also reduced Syk and JNK phosphorylation (S8A Fig), although it did not reach statistical significance (S8B Fig). Thus, Dectin-2 activates both Syk and JNK to transduce signals for inflammasome activation upon interaction with *H*. *capsulatum*. Dectin-1 is also involved in inflammasome activation, although less prominently than Dectin-2, in triggering Syk and JNK signaling pathway.

### Both Dectin-1 and Dectin-2 are important for IL-1β production by CD103^+^ DC in pulmonary *H*. *capsulatum* infection

**Fig 5:**
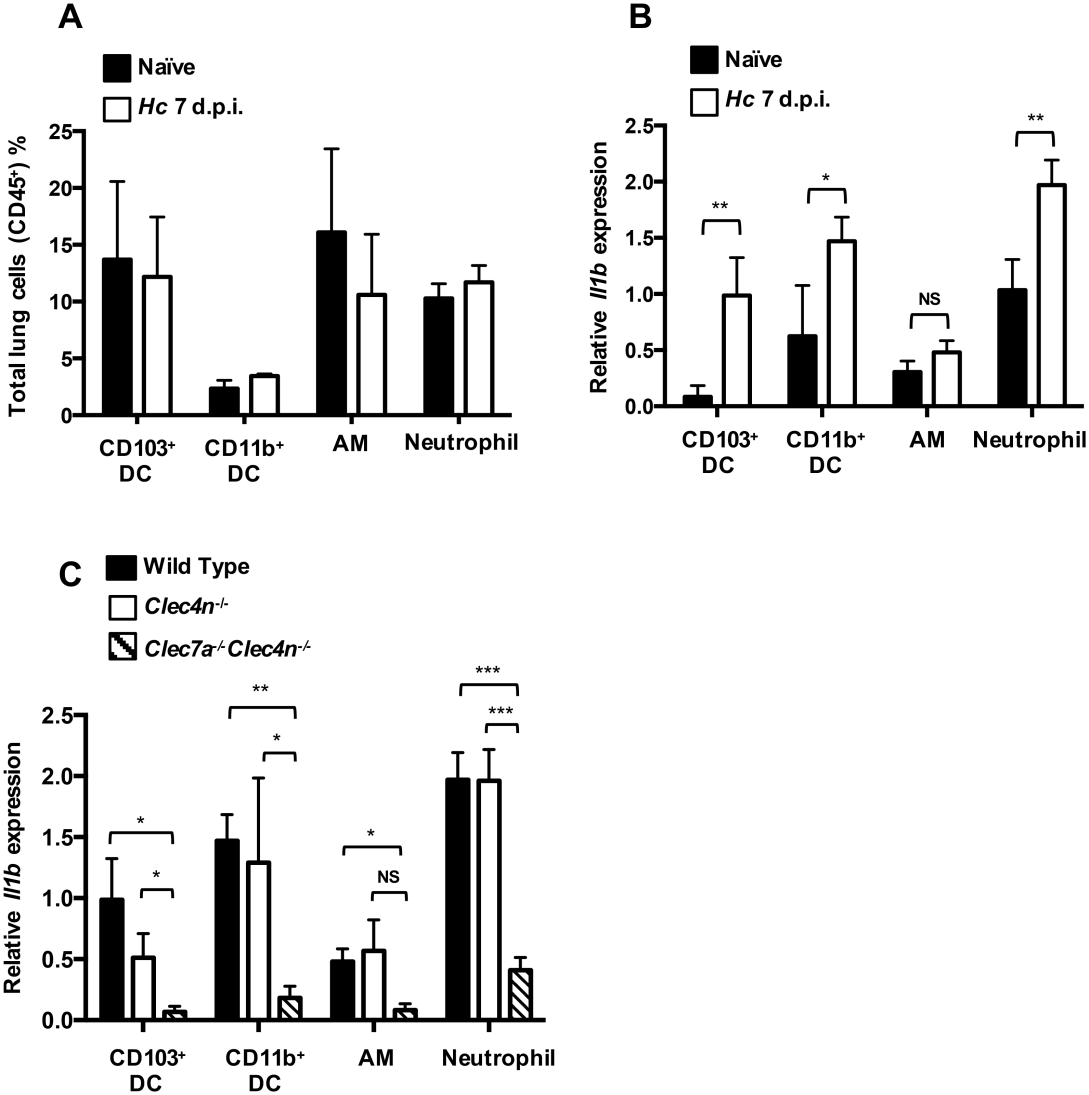
Both Dectin-1 and Dectin-2 are important for IL-1β production by CD103^+^ DC in pulmonary *H*. *capsulatum* infection. (A, B and C) Wild type, *Clec4n*^-/-^ and *Clec7a*^-/-^*Clec4n*^-/-^ mice were intratracheally infected with or without 2.5 × 10^5^ *H*. *capsulatum*. Mice were killed on day 7 after infection. Lungs were digested and CD103^+^ DCs, CD11b^+^ DCs, alveolar macrophages (AM) and neutrophils were sorted based on the following markers: CD103^+^ DCs (CD11c^+^CD103^+^CD11b^-^), CD11b^+^ DCs (CD11c^+^CD103^-^CD11b^+^), alveolar macrophages (Siglec-F^+^F4/80^+^), neutrophils (CD11b^+^Ly6G^+^). Sorted cells were subjected to mRNA extraction. *Il1b* mRNA expression was analyzed by RT-qPCR (n = 3). Error bars indicate standard deviation of the mean. * *p* < 0.05, ** *p* < 0.01, *** *p* < 0.001, NS, not significant [two-way ANOVA with Sidak post-hoc analysis (A & B) or Tukey post-hoc analysis (C)].

We found that CD103^+^ DCs, Siglec-F^+^F4/80^+^ alveolar macrophages and Ly6G^+^CD11b^+^ neutrophils were three major cell populations in the lungs before and after intratracheal *H*. *capsulatum* infection (S9 Fig and Fig 5A). While infection did not change the percentages of CD103^+^ DCs (13–14%), CD11b^+^ DCs (2–3%), alveolar macrophages (11–16%), and neutrophils (10–12%) in the lungs (Fig 5A), there was a 10-fold induction of *Il1b* mRNA in CD103^+^ DCs, 2-fold induction in CD11b^+^ DCs, 1.5-fold induction in alveolar macrophages and 2-fold induction in neutrophils after infection (Fig 5B). These data showed that CD103^+^ DCs are one of the major producers of IL-1β in the lungs upon pulmonary *H*. *capsulatum* infection. Interestingly, the levels of *Il1b* mRNA in CD103^+^ DCs from infected Dectin-2-deficient mice were lower than cells from infected wild type mice although the difference did not reach statistical significance (Fig 5C). Dectin-1 and Dectin-2 double deficiency significantly reduced *Il1b* mRNA expression in all four cell populations (Fig 5C). Together, these data demonstrate that CD103^+^ DC being one of the major sources of IL-1β in pulmonary *H*. *capsulatum* infection, both Dectin-2 and Dectin-1 play an important role in DC response to *H*. *capsulatum*.

### Cathepsin B and K^+^ efflux, but not ROS synthesis, function as signal 2 in *H*. *capsulatum*-stimulated inflammasome activation

**Fig 6:**
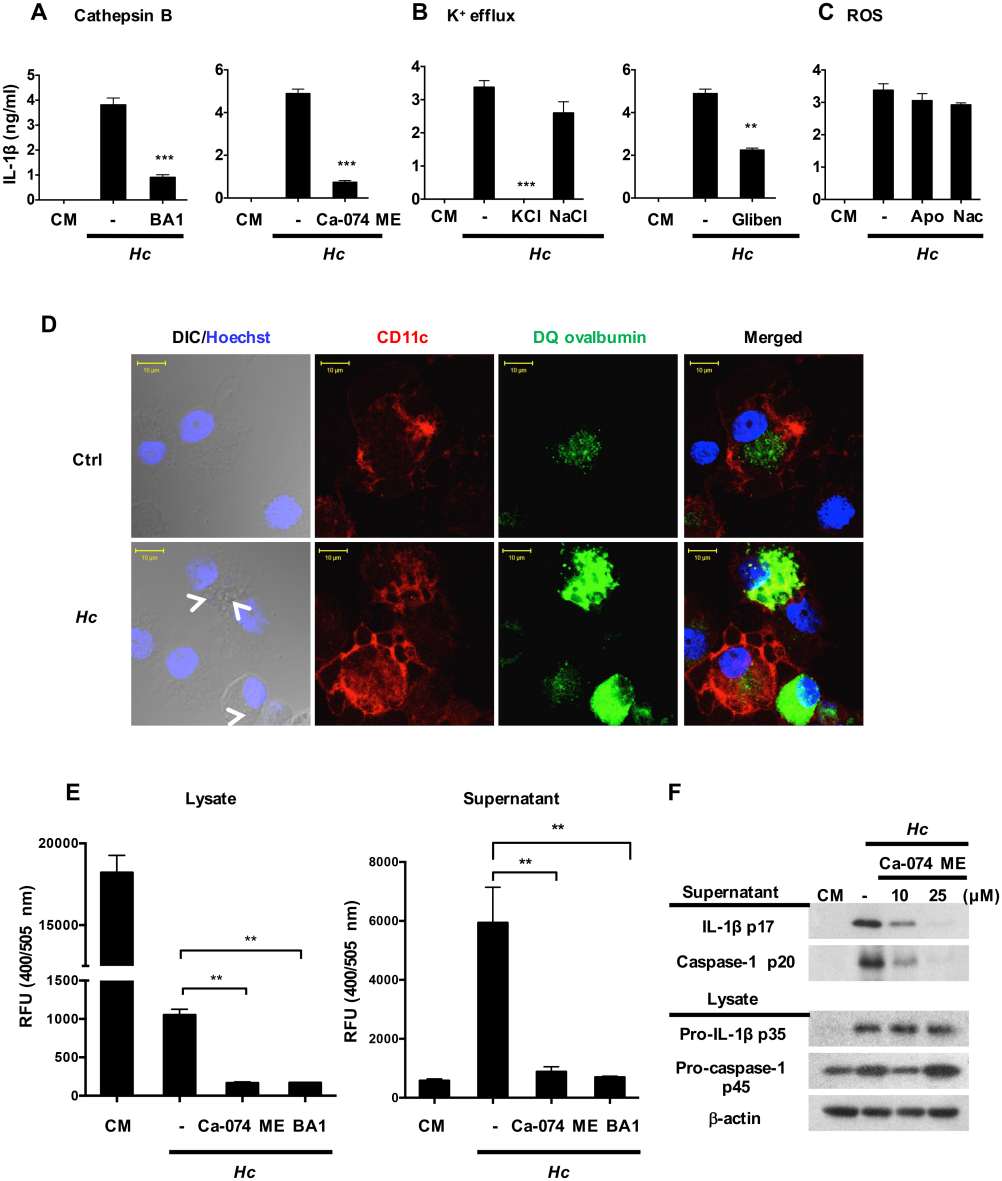
Cathepsin B and K^+^ efflux both function as signals 2 in *H*. *capsulatum*-stimulated inflammasome activation. Wild type BMDCs were pretreated with (A) cathepsin B activity inhibitor (Ca-074 ME, 25 μM), phagosome acidification inhibitor (bafilomycin A1 (BA1), 250 nM), (B) extracellular KCl (50 mM) and NaCl (50 mM), K^+^ channel inhibitor (glibenclamide (Gliben), 50 μM), (C) ROS inhibitor (apocynin (Apo), 10 μM) and ROS scavenger (N-acetyl cysteine (Nac), 20 mM) for 1 h before stimulation with *H*. *capsulatum*. Cell-free supernatants were harvested at 18 h after stimulation. Secreted IL-1β was quantified by ELISA (n = 3). (D) BMDCs were treated with DQ ovalbumin for 1 h before stimulation with (*Hc*) or without (ctrl) *H*. *capsulatum* at MOI of 1. Cells were collected at 1 h after stimulation before cytospun on microscope slides. Cells were fixed and stained for CD11c (red). The nuclei were stained by Hoechst reagent (blue). Cells were viewed under confocal microscope. White arrows point to ingested-*H*. *capsulatum* yeasts in DIC/Hoechst field. (E) BMDCs (2 × 10^6^) were treated with or without Ca-074 ME (25 μM) and bafilomycin A1 (250 nM) before stimulation by *H*. *capsulatum* at MOI of 20 for 18 h. Cell lysates and supernatants were analyzed for cathepsin B activity (n = 3). (F) Wild type BMDCs were treated with Ca-074 ME (10 and 25 μM) before stimulation with *H*. *capsulatum*. Cell lysates and supernatants were analyzed for inflammasome components by Western blotting. Error bars indicate standard deviation of the mean. One representative of three (A, B and C) and two (D, E and F) independent experiments is presented. * *p* < 0.05, ** *p* < 0.01, *** *p* < 0.001 [2-tailed *t*-test (A, B and C); one-way ANOVA with Tuckey post-hoc analysis (E)].

Next, we analyzed the mechanisms that are required for inflammasome activation by *H*. *capsulatum* and addressed the downstream signaling events triggered by Dectin-2. Results showed that blocking cathepsin B activity or K^+^ release, but not inhibition of ROS, reduced the production of IL-1β in BMDC response to *H*. *capsulatum* (Fig 6A, 6B and 6C). We used DQ ovalbumin fluorescence imaging to investigate whether *H*. *capsulatum* induces lysosomal rupture in BMDCs. Without *H*. *capsulatum* stimulation, DQ ovalbumin ingested by BMDCs was located in endosome/lysosome (Fig 6D **upper panel**). After stimulation with *H*. *capsulatum*, endosome/lysosome became enlarged and leaky (Fig 6D **lower panel**). Cathepsin B activity assay also showed that activated cathepsin B was released to culture supernatants after stimulation (Fig 6E). Inhibiting cathepsin B activity or phagosome acidification reduced the release of activated cathepsin B (Fig 6E). In addition, inhibition of cathepsin B activity reduced the secretion of caspase-1 p20 and IL-1β p17, but not the level of pro-IL-1β, confirming the role of cathepsin B as an upstream regulator of signal 2 during inflammasome activation (Fig 6F). Together, these data suggest that uptake of *H*. *capsulatum* induces dendritic cell lysosomal rupture and cathepsin B release that trigger inflammasome activation.

### Dectin-2 and Dectin-1 singly and cooperatively induce cathepsin B release

**Fig 7:**
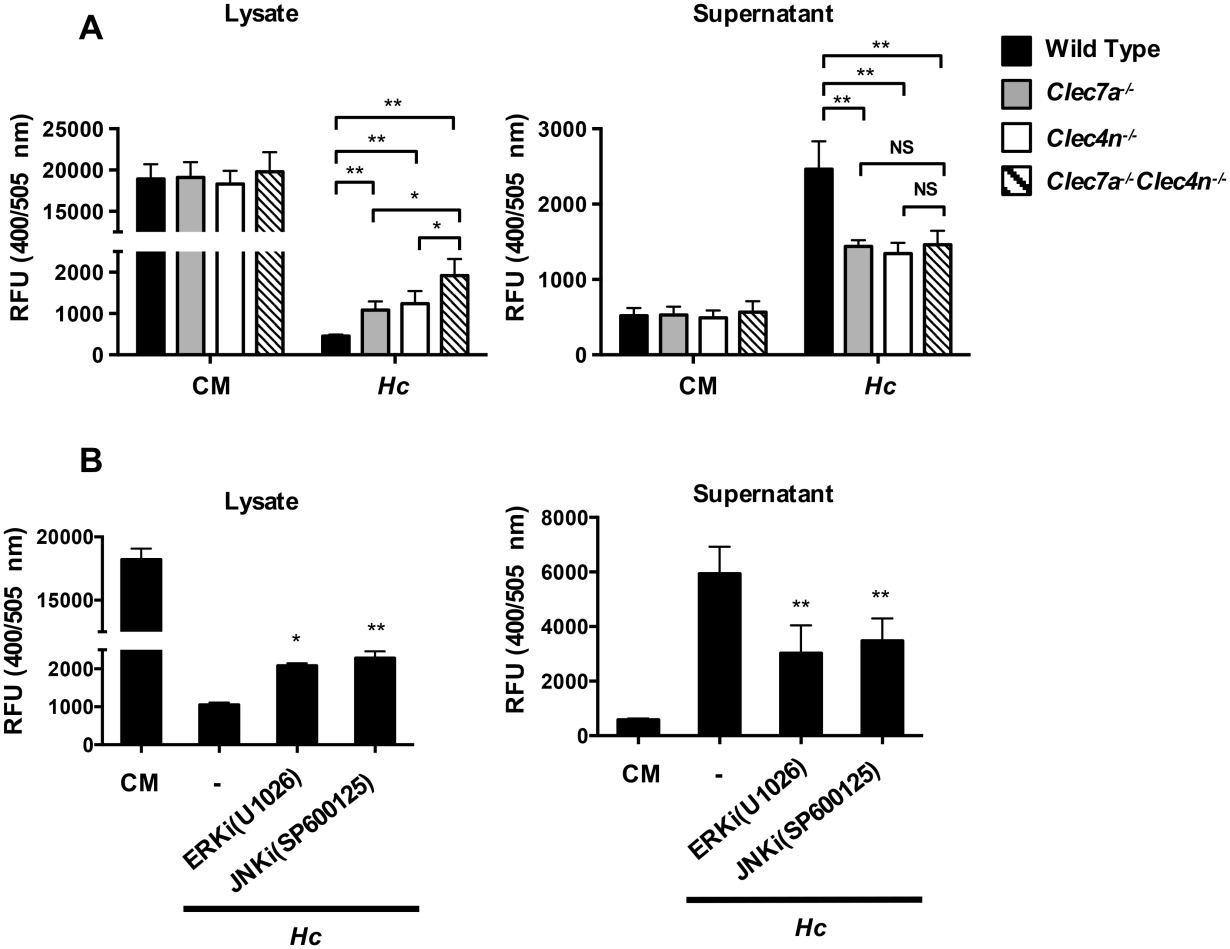
Both Dectin-2 and Dectin-1 and downstream signals regulate dendritic cell release of cathepsin B. BMDCs from wild type, *Clec7a*^-/-^, *Clec4n*^-/-^ and *Clec7a*^-/-^*Clec4n*^-/-^ mice. (A) Cells were stimulated with *H*. *capsulatum*. Cell lysates and supernatants were harvested 18 h later and subjected to cathepsin B activity assay (n = 3). (B) Cells were treated with or without ERK (U1026) and JNK (SP600125) inhibitor before stimulation with *H*. *capsulatum* for 18 h. Cell lysates and supernatants were harvested and subjected to cathepsin B activity assay (n = 3). Error bars indicate standard deviation of the mean. * *p* < 0.05, ** *p* < 0.01, *** *p* < 0.001, NS, not significant [one-way ANOVA with Tuckey post-hoc analysis (A) or Sidak post-hoc analysis (B)].

Cathepsin B activity was quantified in cells with Dectin-1, Dectin-2 single or double deficiency. Data showed that Dectin-1-deficient, Dectin-2-deficient, and double-deficient cells retained activated cathepsin B in the cytosol and double-deficient cells retained significantly more than single-deficient cells (Fig 7A **left panel**). Single- and double-deficient cells reduced the release of activated cathepsin B to culture supernatants although the differences between single- and double-deficient cells were not significant (Fig 7A **right panel**). Additionally, pharmacological inhibition of either ERK or JNK reduced the release of active cathepsin B (Fig 7B). Interestingly, Dectin-2 deficiency, did not make a difference in the kinetics of the drop of intracellular K^+^ levels compared to wild type cells (S10 Fig). These data indicate that, while Dectin-2 does not play a role in K^+^ efflux, Dectin-1 and Dectin-2 singly and cooperatively regulate activated cathepsin B release.

### The viability of *H*. *capsulatum* is critical for signal 2 in inflammasome activation

**Fig 8:**
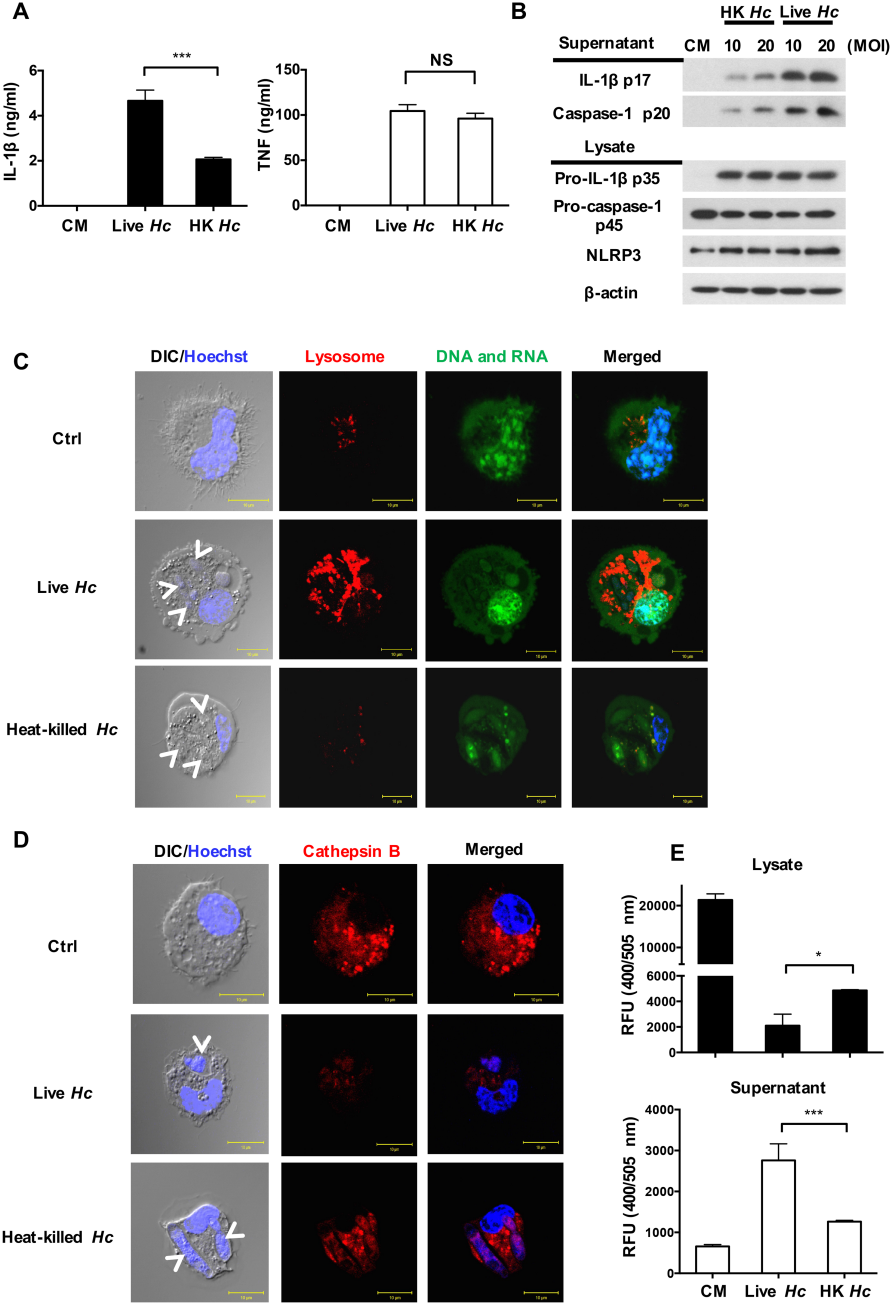
The viability of *H*. *capsulatum* is critical to signal 2 in inflammasome activation. (A and B) BMDCs were stimulated with live or heat-killed *H*. *capsulatum* for 18 h. (A) Secreted IL-1β and TNF in culture supernatants were quantified by ELISA (n = 3). (B) Culture supernatants and cell lysates were subjected to Western blotting. (C and D) Acridine orange and Magic Red^®^ were added 1 h before (C) or after (D) stimulation with live or heat-killed *H*. *capsulatum*. The nuclei were stained by Hoechst reagent. White arrows point to ingested-*H*. *capsulatum* yeasts in DIC/Hoechst field. Cells were viewed under confocal microscope. (E) Collected cell lysates and supernatants from live or heat-killed *H*. *capsulatum* stimulated cells were subjected to cathepsin B activity assay. Error bars indicate standard deviation of the mean. One representative of three (A) or two (B and E) independent experiments is presented. * *p* < 0.05, *** *p* < 0.001 [2-tailed *t*-test (A and E)].

It has been reported that both heat-killed and UV-inactivated *C*. *albicans*, unlike their viable counterparts, fail to induce IL-1β secretion from LPS-primed BMDMs [29]. A separate study showed that heat inactivation and proteinase K digestion of *Schistosoma* egg antigen (SEA) abolish its ability to induce signal 2 [30]. We found that viable *H*. *capsulatum* induced higher levels of IL-1β than their heat-killed counterparts while the viability of the organism did not affect the levels of TNF production (Fig 8A). Western blot analysis also showed that live organism induced higher levels of caspase-1 p20 and IL-1β p17 (Fig 8B) than killed microorganism. It is worth noting that the viability of *H*. *capsulatum* did not affect the levels of pro-IL-1β p35 or NLRP3 (Fig 8B). These data showed that the viability of *H*. *capsulatum* affects signal 2, but not signal 1. Acridine orange was used to analyze lysosome swelling and fluorescent cathepsin B peptide substrate and activity assay to study activated cathepsin B release. Results showed that stimulation with viable but not heat-killed *H*. *capsulatum* led to lysosome swelling (Fig 8C) and cathepsin B release from cytosol (Fig 8D and 8E). Therefore, it appears that viable *H*. *capsulatum* efficiently triggers signal 2 by inducing lysosomal rupture and greater cathepsin B release.

## Discussion

Pathogens interacting with PRRs is the first step in inflammasome activation [28]. *Microsporum canis*, *Malassezia* spp. and *C*. *albicans* recognized by C-type lectin receptors transduce signals to induce NLRP3 inflammasome activation in macrophages and DCs [19, 20, 23]. *M*. *canis* interacting with Dectin-1 induces pro-IL-1β transcription in THP-1 cells [20]. *Malassezia* spp. triggers IL-1β production in human monocyte-derived DCs through Dectin-1 [19]. TLR-2 and Dectin-1 are both involved in induction of pro-IL-1β transcription by hyphal forms of *C*. *albicans* in mouse BMDM [23]. However, Dectin-2 is solely responsible for cytokine induction in mouse BMDC including that of IL-1β by yeast form of *C*. *albicans* [24]. Dectin-2 deficiency affects, but does not completely abolish, cytokine induction by hyphal form of *C*. *albicans* [24]. Ritter *et al*. showed in BMDC that *Schistosoma* egg antigen (SEA) engagement with Dectin-2 provides signal 2 after triggering signal 1 with Pam3Cys for NLRP3 inflammasome [30]. Thus, it appears that the interaction of fungal pathogens with receptors to initiate inflammasome activation is complex. It varies according to cell types, the type of pathogens, the different morphological forms and the triggering of signal 1 or signal 2. Data in this study show that Dectin-2 plays a role as a primary receptor that mediates both signal 1 and 2 for NLRP3 inflammasome in *H*. *capsulatum*-stimulated BMDC. In the presence of Dectin-2, Dectin-1 does not respond to *H*. *capsulatum*. In the absence of Dectin-2, however, recognition of *H*. *capsulatum* by Dectin-1 does take place, although less prominently, and it can trigger both signal 1 and 2.

Triggering Dectin-2 downstream signaling by agonistic anti-Dectin-2 antibody results in phosphorylation of Syk, ERK, p38 and JNK [25]. Stimulation of Dectin-2 by *C*. *albicans* yeasts activates Syk downstream CARD9-dependent MAPKs signaling and cytokine production in BMDC [24, 25]. The hyphal form of *C*. *albicans* activates PLC-γ2 through Dectin-2, and it is critical for ROS production in BMDM [31]. Our finding showing that *H*. *capsulatum* stimulation activates Dectin-2 downstream Syk, ERK, JNK and p38 signaling is consistent with that is reported for *C*. *albicans* [24, 25]. We further demonstrated that Syk deficiency or pharmacological inhibition of ERK and JNK phosphorylation inhibits pro-IL-1β, caspase-1 activation and IL-1β production. It is worth noting that inhibiting p38 significantly increases pro-IL-1β expression, suggesting that p38 negatively regulates *Il1b* gene transcription. In a study of malaria hemozoin stimulation of macrophages, Shio *et al*. showed that both ERK and PI3K are involved in activation of NLRP3 inflammasome through Syk and Lyn kinases, whereas p38 plays no role [32]. Thus, MAPK molecules triggered by different stimuli may have distinct functions even on a specific pathway like that results in IL-1β production. ERK, JNK and p38 are activated upon Dectin-2 engagement with *H*. *capsulatum*. While Syk-JNK/ERK signaling positively regulates NLRP3 inflammasome, Syk-p38 serves as a negative regulator.

Growing evidence shows that Dectin-2 collaborates with Dectin-1 to induce cytokine response upon fungal stimulation [25, 33–35]. Blocking Dectin-2 in Dectin-1-deficient BMDC stimulated with *C*. *albicans* nearly abrogates TNF and IL-10 production compared to wild type and Dectin-1-deficient cells [25]. Blocking both Dectin-1 and Dectin-2 completely abolishes the expression of *Il1b* mRNA in human primary monocyte-derived DC responding to *C*. *albicans* compared to blocking each receptor separately [35]. Dectin-1 and Dectin-2 double-deficient BMDC fails to secrete IL-1β in response to the fungal pathogen *Trichophyton rubrum* [34]. These studies indicate that Dectin-1 and Dectin-2 together mediate cytokine production in response to fungal pathogens. However, the relation between the signaling pathway(s) downstream of Dectin-1 and Dectin-2 has not been fully investigated. Results of our study show a collaborative relationship between Dectin-2 and Dectin-1 in activation of Syk-MAPKs pathway for NLRP3 inflammasome activation. Dectin-2 dominates as a receptor to transduce downstream Syk-JNK signaling in triggering NLRP3 inflammasome even in the presence of Dectin-1. When Dectin-2 is absent, Dectin-1-mediated recognition takes over, although responding less prominently, and activates the same signaling pathway. When both Dectin-2 and Dectin-1 are absent, Syk-JNK signaling becomes almost null. Thus, it appears that while Dectin-2 is the primary receptor that recognizes *H*. *capsulatum*, Dectin-1 takes its place in its absence for triggering Syk-JNK signaling for NLRP3 inflammasome in BMDC response to *H*. *capsulatum*.

Coady *et al*. showed in pulmonary *H*. *capsulatum* infection that IL-1R^-/-^ mice survive intranasal infection with 1.8 × 10^4^ of *H*. *capsulatum* [36]. However, Deepe *et al*. reported that while IL-1R^-/-^ mice survive infection with low dose of *H*. *capsulatum* (1 × 10^4^ and 2 × 10^5^), high dose of *H*. *capsulatum* (2 × 10^6^) causes IL-1R^-/-^ mice to die [22]. These results together demonstrate that IL-1β is protective when mice are challenged with high but not low dose of *H*. *capsulatum*. We observed that when mice were infected with lower dose (2 × 10^6^) of *H*. *capsulatum* (S11 Fig), there was no difference in survival between *Nlrp3*^-/-^ and wild type mice. Infection with high dose of the fungus either intratracheally or intravenously, *Nlrp3*^*-/-*^ mice had significantly less survival than wild type mice (Fig 1J and S12 Fig). Our *in vitro* data show that stimulation of cells with higher MOI of fungus dose-dependently elicits greater inflammasome response. It appears that high dose of *H*. *capsulatum* triggers greater NLRP3 inflammasome response and higher IL-1β production that are protective against lethal *H*. *capsulatum* challenge.

It is reported that CD103^+^ conventional DCs in the lungs produce IFN-I through TLR7/9 upon *H*. *capsulatum* infection [15]. CD103^+^ DC IL-2 response to *A*. *fumigatus* is mediated by Dectin-1 and the downstream Ca^++^-calmodulin-dependent NFAT signaling pathway [37]. We provide evidence to show that CD103^+^ DCs are one of the major producers of IL-1β in the lungs in *H*. *capsulatum* infection. Infection induces upregulation of pro-IL-1β in CD103^+^ and CD11b^+^ DCs as well as in neutrophils. The induction was much higher in CD103^+^ DC than in CD11b^+^ DCs and neutrophils, showing that CD103^+^ DCs are more active in producing IL-1β than other two cell types in response to infection. In addition, Dectin-2 singly and in collaboration with Dectin-1 are involved in CD103^+^ DC IL-1β response. Interestingly, while Dectin-2 single deficiency does not affect IL-1β response in CD11b^+^ DCs, alveolar macrophages and neutrophils, Dectin-2 and Dectin-1 double deficiency almost completely abolish IL-1β production in all cell types. It appears that both Dectin-2 and Dectin-1 are important in host IL-1β response to *H*. *capsulatum* pulmonary infection.

The assembly and activation of canonical NLRP3 inflammasome can be triggered by K^+^ efflux, Ca^++^ uptake, reactive oxygen species and lysosomal protein cathepsin B [5–8, 38]. Both ROS production and K^+^ efflux but not cathepsin B are known to drive NLRP3 inflammasome activation as signal 2 in BMDC response to *C*. *albicans* yeasts and *A*. *fumigatus* conidia [16, 18]. Cathepsin B activity is required, however, for NLRP3 inflammasome activation in BMDM response to hyphal form of *C*. *albicans* [29]. It appears that stimulation of different types of cells by different fungal pathogens employs different pathways to activate signal 2. A number of studies indicate that viable microorganism is required to induce signal 2 [18, 29, 30]. In this study, we show that K^+^ efflux and cathepsin B, but not ROS production (even it was produced in S13 Fig), function as signal 2 for *H*. *capsulatum*-induced NLRP3 inflammasome in BMDC. Our data also show that, although viable and heat-killed *H*. *capsulatum* induce comparable levels of pro-IL-1β, viable *H*. *capsulatum* stimulation leads to production of higher levels of activated caspase-1, more lysosomal swelling and increased cathepsin B release than heat-killed organism. It has been previously shown that human monocyte-derived DC is capable of killing intracellular *H*. *capsulatum* by pronounced phagolysosomal fusion [14] and that phagosomal acidification is an early event preceding lysosomal rupture [6]. We speculate that viable *H*. *capsulatum*-induced lysosomal rupture leading to cathepsin B release takes place after phagolysosomal fusion.

The relationship between receptor(s) and its downstream signaling that mediates signal 2 activation has not been very well-established. Recognition of *C*. *albicans* yeast by Dectin-1 induces ROS production in BMDMs [39]. Hyphal form of *C*. *albicans* triggers Dectin-2 downstream PLCγ-2 signaling that activates ROS production [31]. *C*. *albicans* yeast has also been shown to activate Syk to induce ROS production leading to inflammasome activation in BMDC [16]. Compared to ROS, less is known about the receptor(s) and signaling pathway(s) that lead to cathepsin B release. Shio *et al*. reported that malarial hemozoin activates Syk-mediated cathepsin B activation in THP-1 cells [32]. In an arthritis mouse model, intra-articular injection of zymosan induces cathepsin release in a Dectin-1- and NOD2-dependent manner [40]. Interestingly, cathepsin B release is Syk-dependent in B cell receptor-mediated apoptosis [41]. In this study we provide the first direct evidence that C-type lectins, Dectin-2 and Dectin-1, collaboratively regulate cathepsin B release in BMDC.

**Fig 9:**
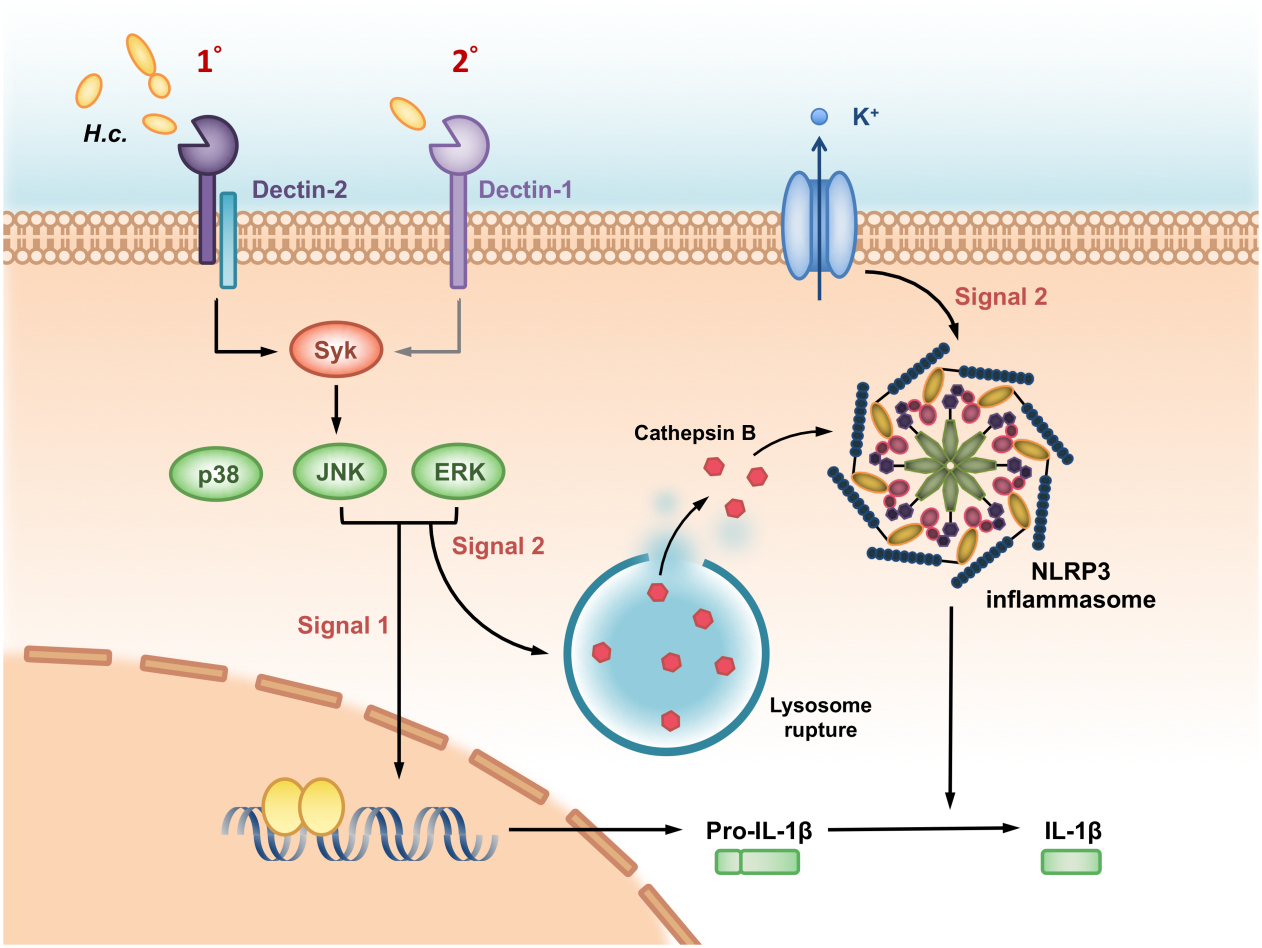
A working model—Dectin-2/Dectin-1 and their downstream signals regulate *H*. *capsulatum*-induced NLRP3 inflammasome activation. Dendritic cells being one of the major IL-1β producers in pulmonary *H*. *capsulatum* infection, the pathogen is recognized preferentially by Dectin-2 and less so by Dectin-1 in dendritic cells. The recognition triggers Syk-JNK signaling pathway that leads to IL-1β production. Syk-JNK signaling provides signal 1 to induce pro-IL-1β synthesis as well as signal 2 to activate cathepsin B release. While K^+^ efflux also functions as signal 2, it is independent of either receptor. Signal 1 and 2 together promote NLRP3 inflammasome assembly and activation for IL-1β release.

Based on our findings demonstrated in this study, we propose a working model as depicted in Fig 9 of the roles of Dectin-2 and Dectin-1 and their downstream signals in *H*. *capsulatum*-induced NLRP3 inflammasome activation in BMDC. Dectin-2 serves as a primary receptor and Dectin-1 as a secondary one in response to *H*. *capsulatum*. Dectin-2 and Dectin-1 downstream signals Syk-JNK trigger both signal 1 to induce pro- IL-1β expression and signal 2 to regulate cathepsin B release for NLRP3 inflammasome activation and IL-1β release. While ROS is not involved, K^+^ efflux also functions as signal 2 but is independent of receptor regulation. Our data are the first to provide insight into the roles of Dectin-2 and Dectin-1 in signaling events for NLRP3 inflammasome activation by *H*. *capsulatum*.

## Materials and methods

### Ethics statement

All animal experiments were undertaken in accordance with the Guidebook for the Care and Use of Laboratory Animals, 3rd Ed., 2007, published by The Chinese-Taipei Society of Laboratory Animal Sciences, approved by the Institutional Animal Care and Use Committee (IACUC, Permit number: 20140533) of National Taiwan University, College of Medicine.

### Mice

C57BL/6 wild type mice (originally from the Jackson Laboratory, Bar Harbor, Maine, USA), *Nlrp3*^*-/-*^ [42], *Clec7a*^*-/-*^ (from Dr. Gordon Brown, University of Cape Town, Cape Town, South Africa), *Clec4n*^*-/-*^ [43], *Clec7a*^-/-^*Clec4n*^-/-^, *Syk*^+/-^ and *FcRγ*^-/-^ (originally from Dr. Clifford Lowell, University of California, San Francisco, CA, USA) were bred and maintained in the Laboratory Animal Center, National Taiwan University, College of Medicine. *Clec7a*^-/-^*Clec4n*^-/-^ mice were obtained from crossing *Clec7a*^-/-^ and *Clec4n*^-/-^ mice. All mice were housed in sterilized cages with sterilized bedding and filter cage tops and were fed with sterilized food and water. Mice at 8–12 weeks of age were used in all experiments.

### Fungus

*H*. *capsulatum* strain 505 was cultured at 37°C on brain heart infusion agar [37 mg/ml; Becton Dickinson Biosciences (BD)] supplemented with 1 mg/ml cysteine (Sigma), 20 mg/ml dextrose (BD) and 20% heat-inactivated certified FBS (Biological Industries). Fresh yeast cell suspensions were prepared in RPMI-1640 medium (Invitrogen) for each experiment. Heat-killed yeast cells were prepared by heating at 65°C water bath for 2 h.

### Cells

Bone marrow cells were flushed from mouse femurs and tibias. Cells were seeded in 24-well culture plate after RBC lysis, cultured in RPMI 1640 complete medium containing 10% heat-inactivated fetal bovine serum (FBS) and 15 ng/ml of GM-CSF. Culture medium was replenished on days 3 and 6. Non-adherent cells were harvested on day 7. About 75–80% of the cells were CD11c^+^ as determined by FACS analysis. Cells were seeded in 96-well plate at 2 × 10^5^ cells /well or in 12-well plate at 2 × 10^6^ cells / well in RPMI 1640 complete medium containing 10% heat-inactivated FBS. Cells were used for experiments 18–24 h later. To obtain Syk^-/-^ cells, Syk^-/-^ embryos were separated from Syk^+/+^ and Syk^+/-^ embryos after crossing Syk^+/-^ mice by their exhibition of severe petechiae and confirmed by genotyping [44]. Single-cell suspensions from fetal liver tissues were cultured in RPMI 1640 complete medium containing GM-CSF for 7 days. Over 80% of the nonadherent cells were CD11C^+^ which were identified as fetal liver-derived dendritic cells (FLDCs).

### Splenic DCs isolation

Splenocytes were collected from wild type, *Nlrp*3^-/-^ and *Clec4n*^-/-^ mice and stained with phycoerythrin (PE)-conjugated anti-MHC class II and allophycocyanin (APC)-conjugated anti-CD11c (eBioscience) antibodies. MHCII^+^CD11c^+^ cells were sorted by FACSAria II cell sorter (BD). Sorted MHCII^+^CD11c^+^ cells were seeded in 96-well plate in RPMI-1640 complete medium containing 10% heat-inactivated FBS and used in experiments immediately.

### *H*. *capsulatum* systemic infection and fungal burden

Mice were intravenously infected with 1 × 10^7^ of *H*. *capsulatum* yeast cells and monitored for 18 days for survival. To determine fungal burdens, spleens were collected on day 11 and spleen homogenates were plated on glucose-peptone agar plates. Mycelial colonies were counted 10 days later.

### Reagents and antibodies

Blocking antibodies anti-Dectin-1 (2A11), anti-Dectin-2 (D2.11E4) and anti-CR3 (5C6) were purchased from Serotec and anti-TLR-2 (6C2), IgG2a (eBR2a) and IgG2b (eB149/10H5) were from eBioscience. PE/Cy7-conjugated anti-CD45 (30-F11), APC-conjugated anti-F4/80 (BM8), Alexa 488-conjugated anti-CD103 (2E7) and Alexa 647-conjugated anti-Ly6G (1A8) were obtained from BioLegend. APC-conjugated anti-CD11c (N418) and PE-conjugated anti-CD11b (M1/70) were from eBioscience and PE-conjugated anti-Siglec-F (E50-2440) from Pharmingen.

Z-YVAD-FMK (Caspase-1 inhibitor) was obtained from BioVision. U1026 (ERK inhibitor), SP600125 (JNK inhibitor), SB203580 (p38 inhibitor), N-acetyl-L-cysteine (ROS scavenger), Apocynin (NADPH-oxidase inhibitor), Ca-074 methyl ester (cathepsin B inhibitor), bafilomycin A1 (phagosomal acidification inhibitor) and glibenclamide (K^+^ channel inhibitor) were obtained from Sigma-Aldrich.

### IL-1β and TNF ELISA assays

BMDCs (2 × 10^5^/well) or splenic DCs (1 × 10^5^/well) were seeded in 96-well plates and cultured overnight before treatment with indicated reagents or blocking antibodies. Live or heat-killed *H*. *capsulatum* (yeast:cell ratio of 10:1 or 20:1) were added 60 min later. Culture supernatants were collected at different time points and stored at -80°C. ELISA kits (eBioscience) were used to quantify IL-1β and TNF in the culture supernatants with 7.8125 pg/ml as the lowest limit of detection.

### Western blotting

BMDCs (2 × 10^6^) were treated with or without blocking antibody for 60 min before the addition of live or heat-killed *H*. *capsulatum*. For inflammasome analysis, cells were cultured in medium containing 0.1% heat-inactivated FBS and for signaling molecule analysis, in medium containing 10% FBS. Cells were detached from the wells and lysed with PhosphoSafe lysis buffer (MERCK) at different time points. Harvested cell-free supernatants were concentrated by 10-fold with Vivaspin 500 (GE Healthcare). Cell lysates were subjected to electrophoresis at 10% (for cell lysates) or 12.5% (for supernatants) SDS polyacrylamide gel and transferred to a 0.45 (for cell lysates) or 0.22 (for supernatants) μm PVDF membrane. The membrane was blocked with 5% non-fat milk and left in buffer containing anti-IL-1β p17 (R&D system), anti-Caspase-1 p20 (Adipogen), anti-NLRP3 (Adipogen), anti-ASC (Adipogen), anti-p-Syk (Abcam), anti-p-ERK (Cell Signaling), anti-p-JNK (Cell Signaling), anti-p-p38 (Cell Signaling) or anti-β-actin (GeneTex) antibody at 4°C overnight. The membrane was washed with TBST before addition of HRP-conjugated anti-goat IgG (1:3000), anti-rabbit (1:20,000) or anti-rat IgG (1:20,000). ECL reagent (PerkinElmer Life Science, Merck Millipore and GE Healthcare) was used for detection.

### ASC pyroptosome detection

BMDCs were seeded in 12-well plates (2 × 10^6^ cells/well) and treated with live *H*. *capsulatum* or LPS (100 ng/ml, 6 h) plus ATP (5 mM, 30 minutes) for 18 h. Cells were centrifuged at 4500 rpm for 5 min. Cell pellets were resuspended in cold 0.3 ml buffer A (20 mM HEPES-KOH, pH 7.5, 10 mM KCl, 1.5 mM MgCl_2_, 1 mM EDTA, 1 mM EGTA, 320 mM sucrose) and protease inhibitor cocktail. Cell lysate was obtained after passing the suspension through 29-G syringe for 15 times. Cell lysates were spun at 4500 rpm for 8 min to remove contaminating nuclei, unlysed cells and *H*. *capsulatum*. Supernatants were diluted with 1 volume of CHAPS buffer (20 mM HEPES-KOH, pH 7.5, 5 mM MgCl_2_, 0.5 mM EGTA and 0.1% CHAPS) to lyse residual organelles before centrifugation at 8000 rpm for 8 min. After centrifugation, supernatants were discarded and the pellets were treated with disuccinimidyl suberate (DSS, 2 mM, Sigma-Aldrich) for 30 min at room temperature. The cross-linked pellets were re-suspended in 40 μl SDS sample buffer and proteins were separated on 12.5% SDS polyacrylamide gel followed by immunoblotting with anti-ASC antibody (Adipogen) as previously described [45]

### Phagocytosis assay

The detailed methodology has been previous described [46]. BMDCs (2 × 10^6^) from wild type, *Clec7a*^-/-^ and *Clec4n*^-/-^ mice were seeded in 12-well plates. The plates were cooled on ice for 20 min before the addition of FITC-labeled *H*. *capsulatum* at MOI of 20. After 60 min cooling on ice, the plates were moved to 37°C CO_2_ incubator for 60 min. Cells were collected and quenched FITC-labeled yeast by trypan blue for 5 min. After washing with dPBS, cells were fixed with 1% paraformaldehyde. The phagocytosis rate was determined by flow cytometry.

### Isolation of lung cells

Wild type, *Clec4n*^-/-^ and *Clec7a*^-/-^*Clec4n*^-/-^ mice were intratracheally infected with 2.5 × 10^5^ *H*. *capsulatum*. Lungs were collected on day 7 after infection and digested with 0.15 mg/ml of Liberase TM (Roche) at 37°C for 30 min on a rotatory agitator. Cell pellets were resuspended in 45% Percoll (GE Healthcare) and overlaid on 81% Percoll. After centrifugation, cells at interface (45/81% Percoll) were harvested and stained with antibodies before sorting by FACSAria II cell sorter (BD). CD45^+^ cells were sub-gated to sort out different cell populations: CD103^+^ DCs (CD11c^+^CD103^+^CD11b^-^), CD11b^+^ DCs (CD11c^+^CD103^-^CD11b^+^), alveolar macrophages (Siglec-F^+^F4/80^+^), neutrophil (CD11b^+^Ly6G^+^).

### RT-qPCR

Splenic DCs and lung cells RNA was extracted by using Quick-RNA^™^ MiniPrep (Zymo Research). cDNA was synthesized in reaction mixture including RNA, 5× first-strand buffer, DTT (0.1M), dNTP (10mM), random primers (50 μM), SuperScript III Reverse Transcriptase (Invitrogen) and RNaseOUT^™^ recombinant ribonuclease inhibitor (Invitrogen) in DEPC-H_2_O.

Quantitative real-time PCR was performed by using ABI7900 Real-time PCR detection system in total volume of 10 μl per reaction, containing 2 μl of cDNA with 0.2 μM forward and reverse primers in 8 μl Fast SYBR Green Master Mix (Applied Biosystems). *Il1b* mRNA was normalized against *Actb* mRNA. Primers for *Il1b* are 5’-GAA CTC AAC TGT GAA ATG CCA CC-3’(forward) and 5’-CCA CAG CCA CAA TGA GTG ATA CT-3’(reverse) and for *Actb* 5’-TGT ATG AAG GCT TTG GTC TCC CT-3’ (forward) and 5’-AGG TGT GCA CTT TTA TTG GTC TCA A-3’ (reverse).

### Lysosomal rupture

BMDCs (1 × 10^5^) were seeded in 96-well plate and treated with 10 μM of DQ ovalbumin (Sigma-Aldrich) for 1 h before stimulation with or without *H*. *capsulatum* at MOI of 1. Cells were collected 1 h later and cytospun on microscope slides. The cytospun cells were fixed in 4% paraformaldehyde, blocked with 5% heat-inactivated FBS and stained with APC-conjugated anti-CD11c antibody (eBioscience). Hoechst 33258 was used to stain cell nuclei. The slides were viewed under Zeiss LSM 510 META Confocal Microscope.

### Cathepsin B activity assay

BMDCs (2 × 10^6^) were cultured in 12-well plate with phenol red-free RPMI complete medium (Invitrogen) and stimulated with or without *H*. *capsulatum* at MOI of 20. Cells and culture supernatants were collected separately 18 h after stimulation. Cell-free supernatants were concentrated 10-fold by Vivaspin 500 (GE Healthcare). Cells were lysed with cathepsin B lysis buffer (Abcam, ab65300). Both cell lysates and concentrated supernatants were loaded unto 96-well plate (solid black, Corning) before addition of CB reaction buffer. CB substrate Ac-RR-AFC (final concentration of 200 μM) was added and incubated at 37°C for 1 h. Samples were read in SpectraMax M5 (Molecular Devices) at 400 nm excitation and 505 nm emission wavelengths.

### Intracellular K^+^ concentration

BMDCs (2 × 10^5^) were seeded in 96-well plate (black with clear flat-bottom tissue culture plate, Corning) and were loaded with 2 μM potassium-binding benzofuran isophthalate-AM (PBFI/AM, Molecular Probes, Thermo Fisher Scientific) in phenol red-free RPMI medium (Invitrogen) in the presence of 0.05% (w/w) Pluronic F-127 (Sigma-Aldrich) at 25°C in the dark for 60 min. After one wash, cells were stimulated with *H*. *capsulatum* at MOI of 1. Fluorescence intensity of PBFI (excitation wavelength 340 nm, emission wavelength 500 nm) was recorded every min by SpectraMax M5 while the culture was maintained in 37°C. Stimulation with ATP (5 mM) was used as a positive control.

### Staining for lysosome and activated cathepsin B

BMDCs (2 × 10^5^) in phenol red-free RPMI complete medium were seeded in 96-well plate. Acridine orange was added to medium 1 h before and Magic Red^®^ (ImmunoChemistry Technologies) added 1 h after addition of live or heat-killed *H*. *capsulatum* stimulation. Hoechst 33258 was added 3 h later. Cells were washed and cell suspension was placed onto microscope slide for viewing under confocal microscope. Acridine orange bound to nuclear and cytosolic DNA and RNA emits green fluorescence (excitation wavelength 480 nm, emission wavelength 550 nm). Due to its cationic nature, acridine orange emits red fluorescence (excitation wavelength 550 nm, emission wavelength 620 nm) in acidic compartments such as lysosome. Active cathepsin B reacts with Magic Red^®^ substrates and generates a strong red fluorescence (excitation wavelength 592 nm, emission wavelength 628 nm).

### ROS production

BMDCs (1.2 × 10^6^) were incubated in phenol red-free HBSS containing 10 μM CM-H_2_DCFDA (Thermo Fisher Scientific) for 30 min at 37°C. After replenishment with fresh phenol red-free RPMI 1640 complete medium, cells were stimulated with *H*. *capsulatum* (MOI = 20). Oxidative DCF was analyzed by flow cytometry.

### Statistics

Differences between treatment groups were analyzed with the student 2-tailed *t* test (comparing two group) and one-way (comparing multiple groups with one control) or two-way (comparing multiple groups with two control groups) ANOVA followed by Tukey or Sidak post-hoc test. Log-rank test was used to analyze % survival. All statistics analyses were calculated by Prism 6 software. The level of statistical significance was defined as *p*<0.05. All results were expressed as mean ± standard deviation of the mean.

## Supporting information

S1 Fig The absence of NLRP3 does not affect *H*. *capsulatum*-induced TNF production in dendritic cells. (A) BMDCs and (B) sorted splenic DCs from wild type and NLRP3-deficient (*Nlrp3*^*-/-*^) mice were stimulated with *H*. *capsulatum* at MOI of 10 and 20 for 18 h. Stimulation with LPS (500 ng/ml, 6 h) plus ATP (5 mM, 30 minutes) (L+A) was used as a positive control for TNF induction. (A and B) TNF in the supernatants were quantified by ELISA (n = 3). Error bars indicate standard deviation of the mean. One representative of three independent experiments is presented. [2-tailed *t*-test].
(TIF)

S2 Fig *FcRγ*^*-/-*^ BMDCs produce lower levels of IL-1β and caspase-1 p20 upon *H*. *capsulatum* stimulation. (A and B) BMDCs from wild type and Fc receptor γ chain-deficient (*FcRγ*^*-/-*^) mice were stimulated with or without *H*. *capsulatum*. (A) IL-1β in cell-free supernatants were quantified by ELISA (n = 3). (B) Cell lysates and supernatants were subjected to Western blotting. Error bars indicate standard deviation of the mean. ** *p* < 0.01 [2-tailed *t*-test (A)].
(TIF)

S3 Fig Syk deficiency reduces expression of pro-caspase-1 p45 after *H*. *capsulatum* stimulation. Relative intensity of pro-caspase-1 p45 were quantified by ImageJ (n = 3) from data in Fig 3D. Data were pooled from three independent experiments. * *p* < 0.05 [one-way ANOVA with Tuckey post-hoc analysis].
(TIF)

S4 Fig Surface expression of Dectin-1 and Dectin-2 on wild type, Dectin-1-deficient and Dectin-2-deficient BMDCs. Cells from wild type, Dectin-1-deficient (*Clec7a*^-/-^), and Dectin-2-deficient (*Clec4n*^-/-^) mice were stained with APC-anti-CD11c antibody, purified-antibody against Dectin-1 and Dectin-2 and Alexa 488-goat anti-rat IgG. IgG2a and IgG2b were used as isotype controls. Histograms show the fluorescence intensity of each receptor on CD11c^+^ cells.
(TIF)

S5 Fig Neither Dectin-1 nor Dectin-2 is involved in phagocytosis of *H*. *capsulatum*. BMDCs from wild type, Dectin-1-deficient (*Clec7a*^-/-^), and Dectin-2-deficient (*Clec4n*^-/-^) mice were allowed to take up FITC-labeled *H*. *capsulatum* at MOI of 20. After cold treatment at 4°C for 1 h, followed by incubation at 37°C for 1 h, cells were treated with trypan blue to quench uningested yeasts. Percentages of CD11c^+^ cells taking up *H*. *capsulatum* were analyzed by flow cytometry. Error bars indicate standard deviation of the mean. [one-way ANOVA with Tuckey post-hoc analysis].
(TIF)

S6 Fig The roles of Dectin-2 and Dectin-1 in inflammasome activation. (A and B) BMDCs from wild type, Dectin-2-deficient (*Clec4n*^-/-^) and Dectin-1-deficient (*Clec7a*^-/-^) mice were pretreated with or without anti-Dectin-1 or -Dectin-2 blocking antibody (2 μg/ml) for 1 h before stimulation with *H*. *capsulatum*. Cell culture supernatants were collected at 18 h after stimulation. (A) Secreted IL-1β was quantified by ELISA (n = 5). (B) Cell-free supernatants and cell lysates were subjected to Western blotting analysis. IgG2a and IgG2b were used as isotype controls. Error bars indicate standard deviation of the mean. One representative of three (A) or two (B) independent experiments is presented. * *p* < 0.05, ** *p* < 0.01, *** *p* < 0.001 [two-way ANOVA with Tukey post-hoc analysis (A)].
(TIF)

S7 Fig Dectin-2 and Dectin-1 double deficiency completely abrogates Syk-JNK signaling. BMDCs from wild type, Dectin-1 (*Clec7a*^*-/-*^) and Dectin-2 (*Clec4n*^*-/-*^) single-deficient and double-deficient (*Clec7a*^-/-^*Clec4n*^-/-^) mice were stimulated with *H*. *capsulatum*. Collected cell lysates were analyzed for MAPK signaling molecules by Western blotting. One representative of three independent experiments is presented. Relative intensity of phosphorylated MAPK molecules were quantified by ImageJ. Error bars indicate standard deviation of the mean. (n = 3) * *p* < 0.05, ** *p* < 0.01, *** *p* < 0.001 [one-way ANOVA with Tukey post-hoc analysis and 2-tailed *t*-test (B)].
(TIF)

S8 Fig The effect of reciprocal blocking of Dectin-1 and Dectin-2 in single receptor deficient cells on signal pathway. (A) BMDCs from wild type, Dectin-2-deficient (*Clec4n*^-/-^) and Dectin-1-deficient (*Clec7a*^-/-^) mice were pretreated with Dectin-1 and Dectin-2 blocking antibody, respectively, before stimulation with *H*. *capsulatum*. Collected cell lysates were analyzed for MAPK signaling molecules by Western blotting. One representative of three independent experiments is presented. (B) Relative intensity of phosphorylated MAPK molecules were quantified by ImageJ (n = 3). IgG2a and IgG2b were used as isotype controls. Error bars indicate standard deviation of the mean. * *p* < 0.05, ** *p* < 0.01, *** *p* < 0.001, NS, not significant [two-way ANOVA with Tukey post-hoc analysis and 2-tailed *t*-test (B)].
(TIF)

S9 Fig Flow cytometric analysis of cell populations in the lungs. The lung cell population was determined by flow cytometry based on the following gating strategy: Viable cells were selected by gating out debris. CD45^+^CD11c^+^CD103^+^CD11b^-^ cells are designated as CD103^+^ DC and CD45^+^CD11c^+^CD103^-^CD11b^+^ cells as CD11b^+^ DC, Siglec-F^+^F4/80^+^ cells as alveolar macrophages, and CD11b^+^Ly6G^+^ as neutrophils.
(TIF)

S10 Fig *H*. *capsulatum*-induced K^+^ efflux is independent of Dectin-2. BMDCs (2 × 10^5^) from wild type and *Clec4n*^-/-^ mice were incubated with potassium-sensitive probe PBFI/AM (2 μM) in the presence of Pluronic F-127 (0.05%) at room temperature in the dark for 60 min. After one wash, cells were stimulated with *H*. *capsulatum* at MOI of 1. Cells stimulation with ATP at 5 mM was used as a positive control for induction of K^+^ efflux. Fluorescence intensity ratio of PBFI (excitation wavelength 340 nm, emission wavelength 500 nm) was recorded every min for 30 mins. One representative of two independent experiments is presented.
(TIF)

S11 Fig *Nlrp3*^-/-^ mice survive low dose of intravenous *H*. *capsulatum* infection. WT and *Nlrp3*^-/-^ mice were intravenously infected with *H*. *capsulatum* (2 × 10^6^). Survival was analyzed by log-rank test.
(TIF)

S12 Fig *Nlrp3*^-/-^ mice succumb to high dose of intratracheal *H*. *capsulatum* infection. WT and *Nlrp3*^-/-^ mice were intratracheally infected with *H*. *capsulatum* (1 × 10^7^). Survival was analyzed by log-rank test. * *p* < 0.05.
(TIF)

S13 Fig *H*. *capsulatum* induces ROS production in BMDC. BMDCs (1.2 × 10^6^) from wild type mice were incubated with 10 μM of CM-H_2_DCFDA for 30 min before stimulation with or without *H*. *capsulatum*. The levels of ROS production are shown as mean florescence intensity (MFI) of oxidized DCF fluorescence (n = 3). The MFI at zero minute represents value of the control without stimulation. One representative of two independent experiments is shown.
(TIF)
