# Supplementary figures and images for: Dectin-2 is a primary receptor for NLRP3 inflammasome activation in dendritic cell response to Histoplasma capsulatum
Source: PLoS Pathog. 2017 Jul 3;13(7):e1006485. doi: 10.1371/journal.ppat.1006485 (PMC5510910; doi:10.1371/journal.ppat.1006485)

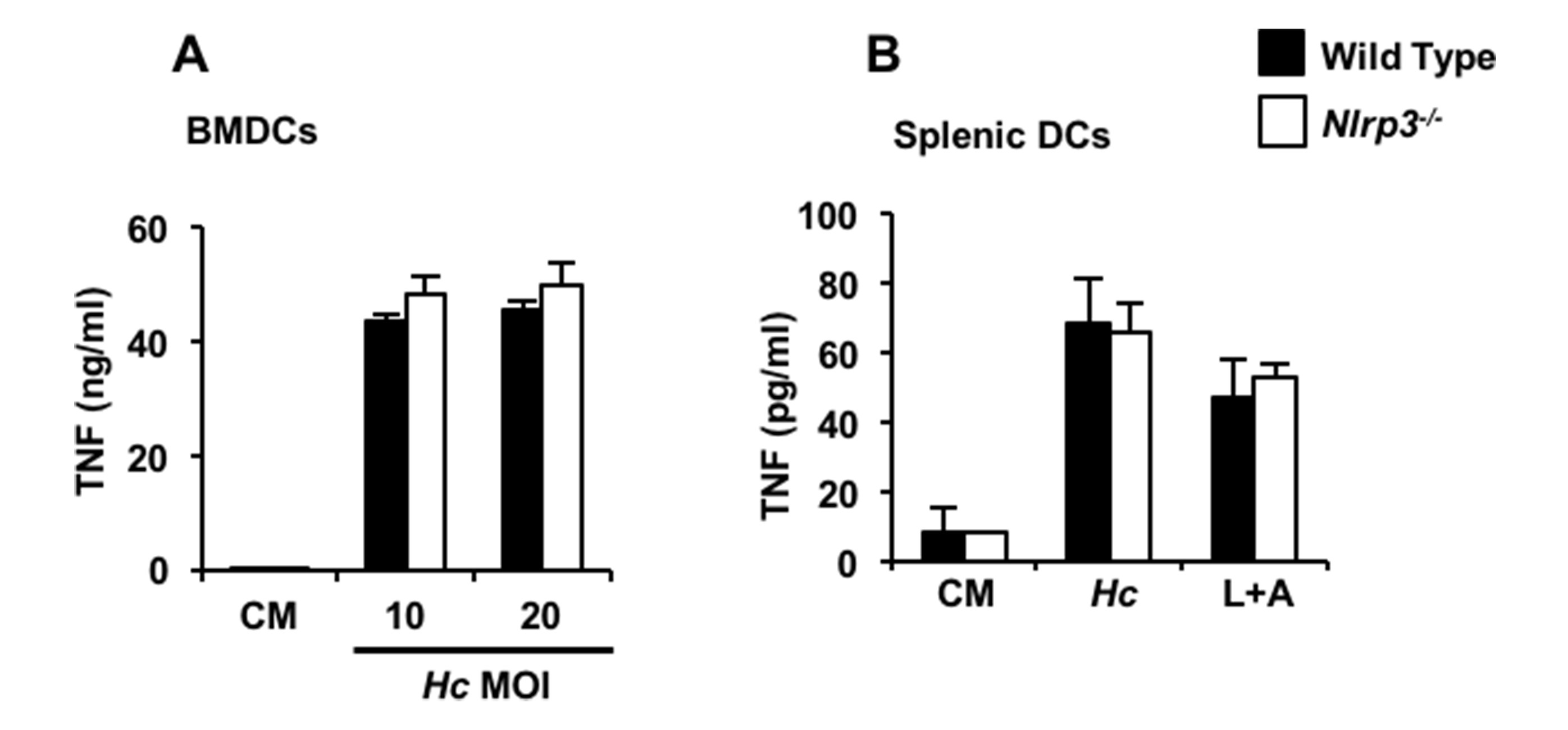

Supplement: S1 Fig — (A) BMDCs and (B) sorted splenic DCs from wild type and NLRP3-deficient (Nlrp3-/-) mice were stimulated with H. capsulatum at MOI of 10 and 20 for 18 h. Stimulation with LPS (500 ng/ml, 6 h) plus ATP (5 mM, 30 minutes) (L+A) was used as a positive control for TNF induction. (A and B) TNF in the supernatants were quantified by ELISA (n = 3). Error bars indicate standard deviation of the mean. One representative of three independent experiments is presented. [2-tailed t-test]. (TIF) [file ppat.1006485.s001.tif]

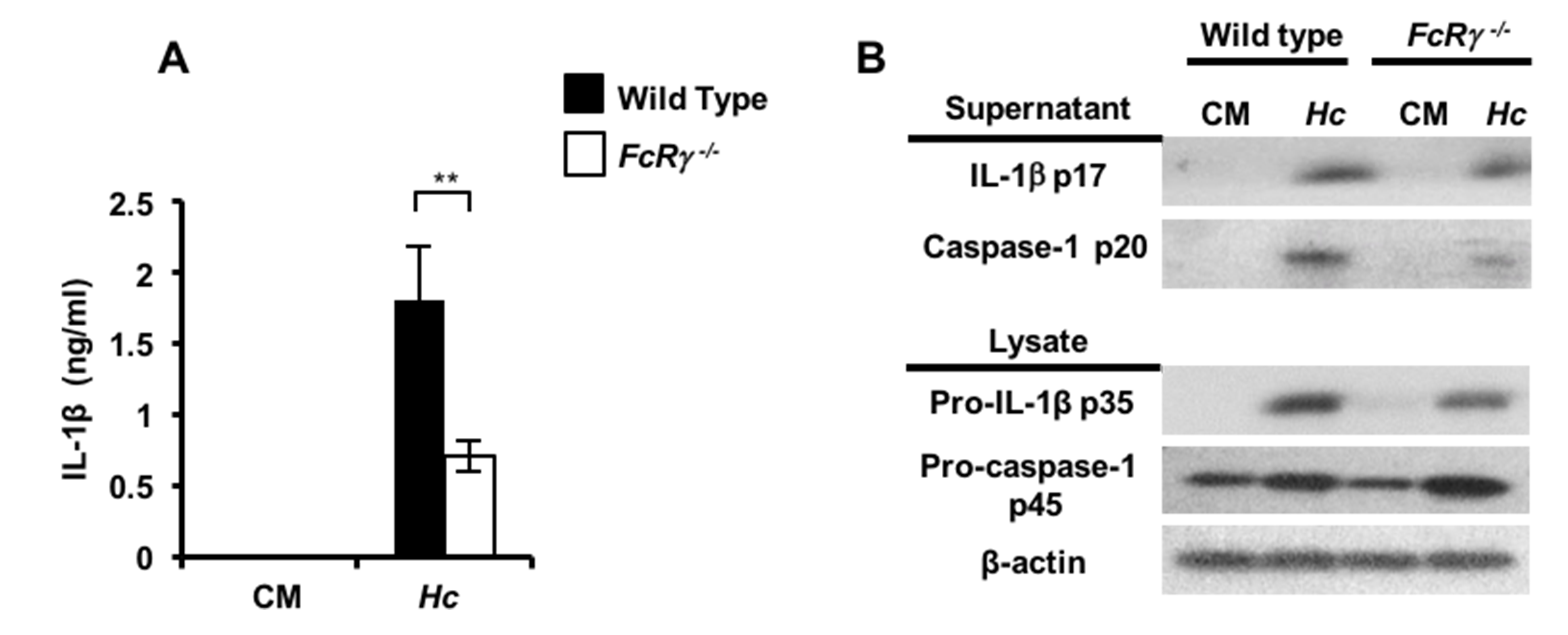

Supplement: S2 Fig — (A and B) BMDCs from wild type and Fc receptor γ chain-deficient (FcRγ-/-) mice were stimulated with or without H. capsulatum. (A) IL-1β in cell-free supernatants were quantified by ELISA (n = 3). (B) Cell lysates and supernatants were subjected to Western blotting. Error bars indicate standard deviation of the mean. ** p < 0.01 [2-tailed t-test (A)]. (TIF) [file ppat.1006485.s002.tif]

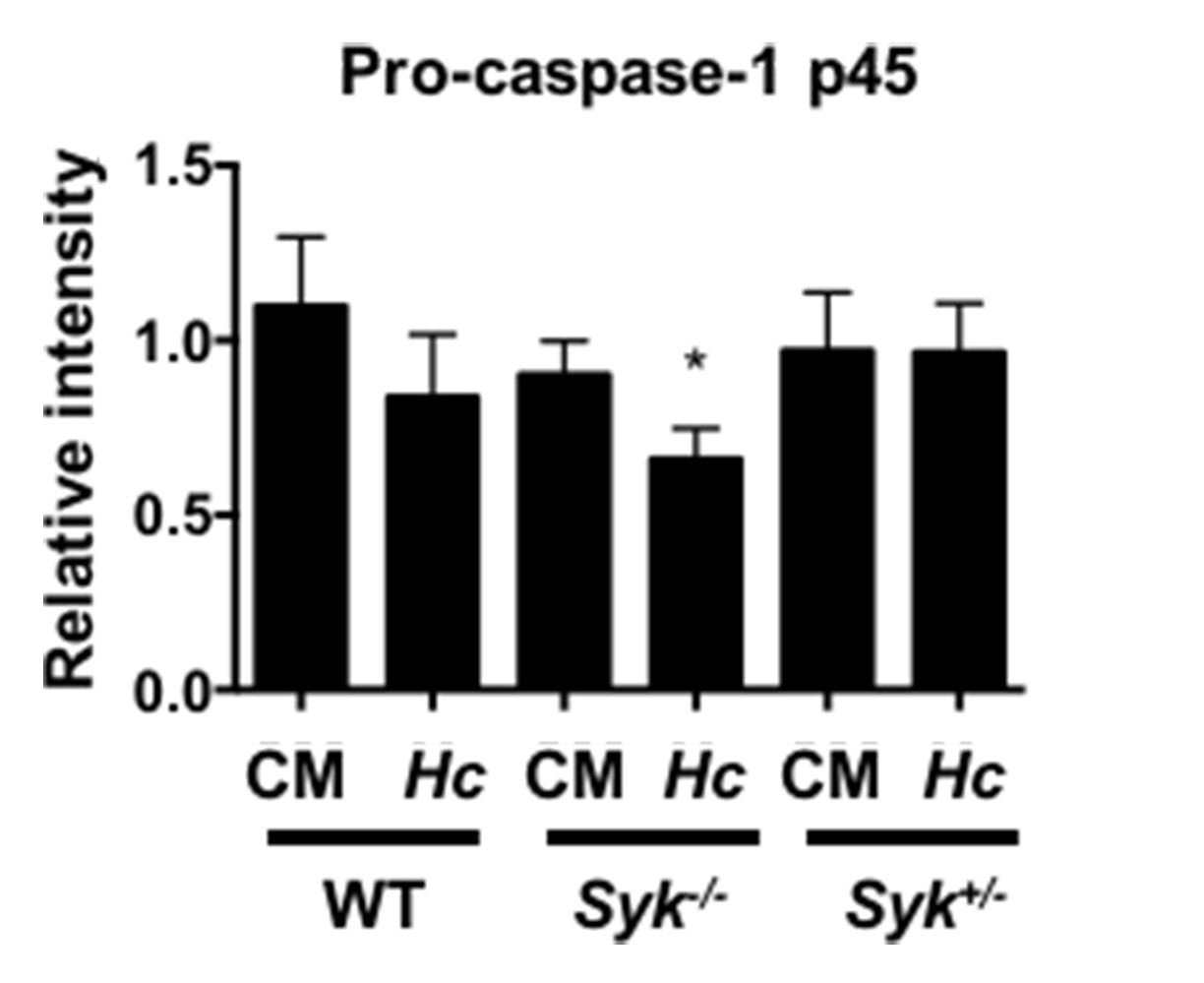

Supplement: S3 Fig — Relative intensity of pro-caspase-1 p45 were quantified by ImageJ (n = 3) from data in Fig 3D. Data were pooled from three independent experiments. * p < 0.05 [one-way ANOVA with Tuckey post-hoc analysis]. (TIF) [file ppat.1006485.s003.tif]

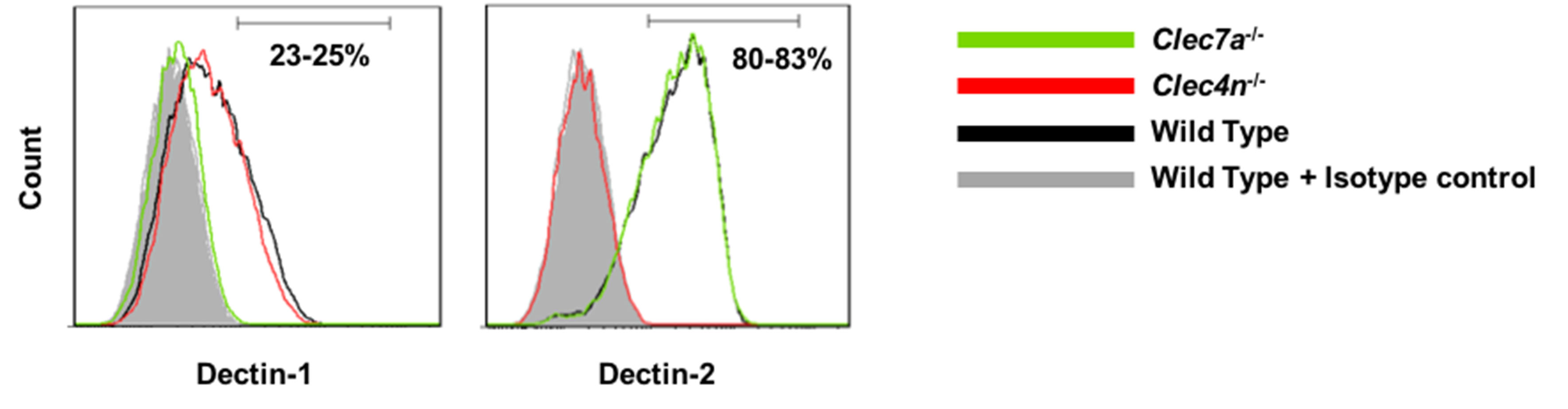

Supplement: S4 Fig — Cells from wild type, Dectin-1-deficient (Clec7a-/-), and Dectin-2-deficient (Clec4n-/-) mice were stained with APC-anti-CD11c antibody, purified-antibody against Dectin-1 and Dectin-2 and Alexa 488-goat anti-rat IgG. IgG2a and IgG2b were used as isotype controls. Histograms show the fluorescence intensity of each receptor on CD11c+ cells. (TIF) [file ppat.1006485.s004.tif]

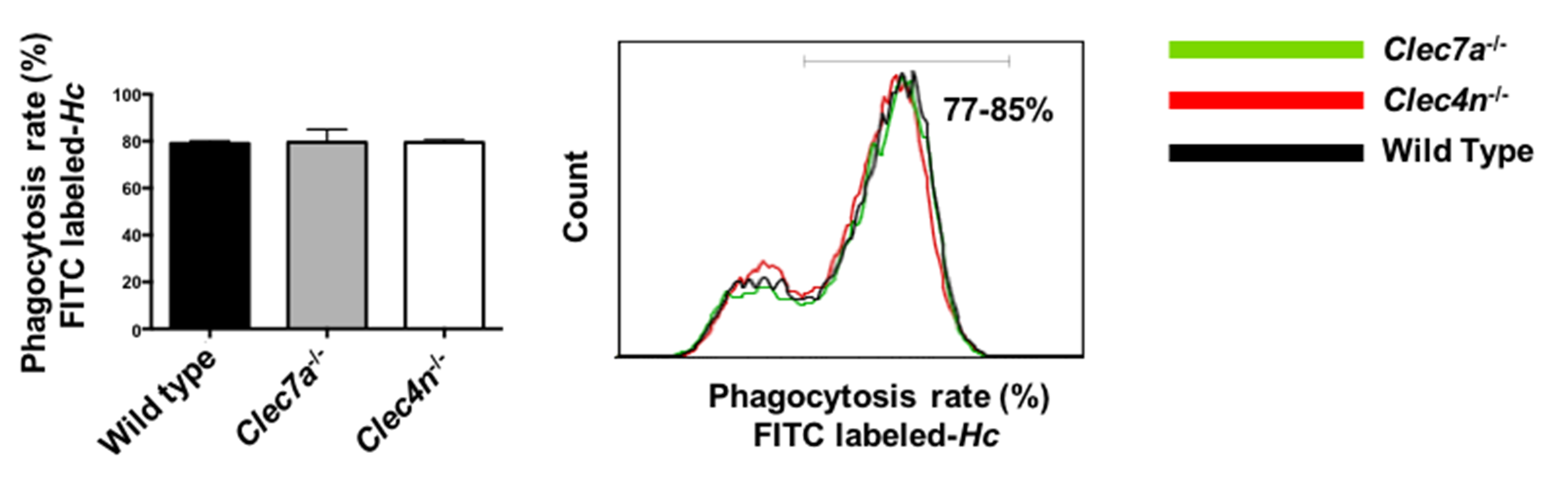

Supplement: S5 Fig — BMDCs from wild type, Dectin-1-deficient (Clec7a-/-), and Dectin-2-deficient (Clec4n-/-) mice were allowed to take up FITC-labeled H. capsulatum at MOI of 20. After cold treatment at 4°C for 1 h, followed by incubation at 37°C for 1 h, cells were treated with trypan blue to quench uningested yeasts. Percentages of CD11c+ cells taking up H. capsulatum were analyzed by flow cytometry. Error bars indicate standard deviation of the mean. [one-way ANOVA with Tuckey post-hoc analysis]. (TIF) [file ppat.1006485.s005.tif]

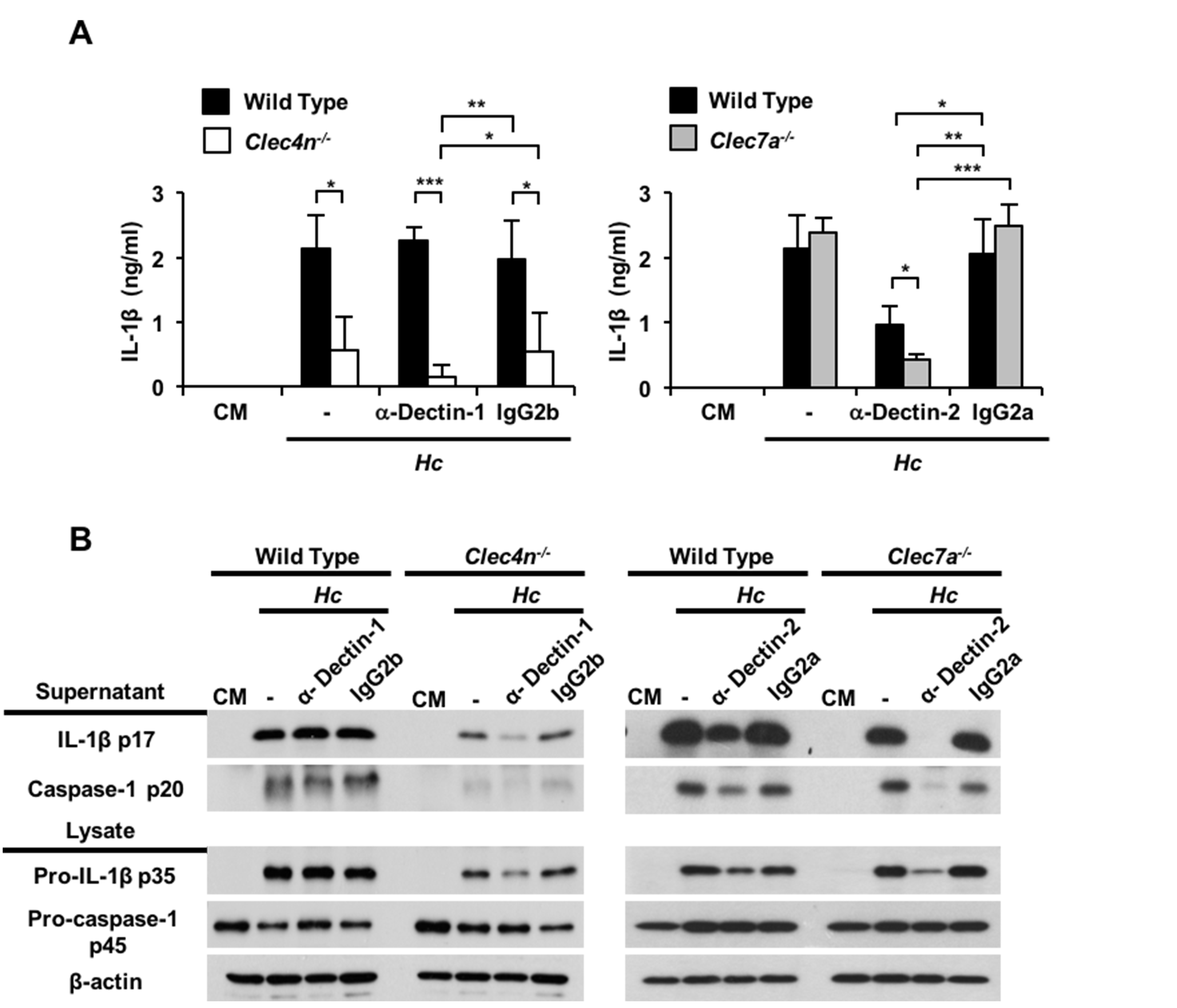

Supplement: S6 Fig — (A and B) BMDCs from wild type, Dectin-2-deficient (Clec4n-/-) and Dectin-1-deficient (Clec7a-/-) mice were pretreated with or without anti-Dectin-1 or -Dectin-2 blocking antibody (2 μg/ml) for 1 h before stimulation with H. capsulatum. Cell culture supernatants were collected at 18 h after stimulation. (A) Secreted IL-1β was quantified by ELISA (n = 5). (B) Cell-free supernatants and cell lysates were subjected to Western blotting analysis. IgG2a and IgG2b were used as isotype controls. Error bars indicate standard deviation of the mean. One representative of three (A) or two (B) independent experiments is presented. * p < 0.05, ** p < 0.01, *** p < 0.001 [two-way ANOVA with Tukey post-hoc analysis (A)]. (TIF) [file ppat.1006485.s006.tif]

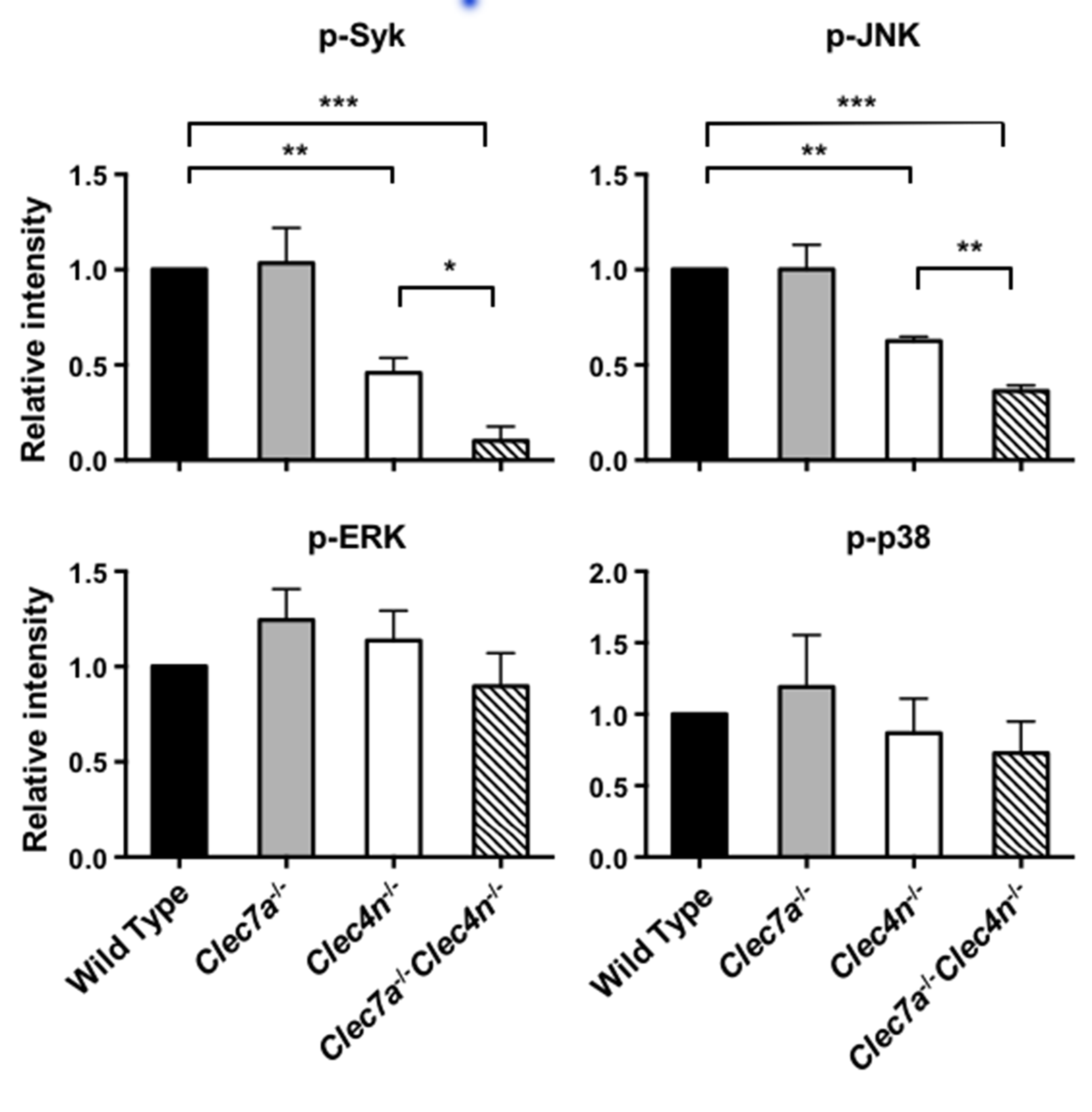

Supplement: S7 Fig — BMDCs from wild type, Dectin-1 (Clec7a-/-) and Dectin-2 (Clec4n-/-) single-deficient and double-deficient (Clec7a-/-Clec4n-/-) mice were stimulated with H. capsulatum. Collected cell lysates were analyzed for MAPK signaling molecules by Western blotting. One representative of three independent experiments is presented. Relative intensity of phosphorylated MAPK molecules were quantified by ImageJ. Error bars indicate standard deviation of the mean. (n = 3) * p < 0.05, ** p < 0.01, *** p < 0.001 [one-way ANOVA with Tukey post-hoc analysis and 2-tailed t-test (B)]. (TIF) [file ppat.1006485.s007.tif]

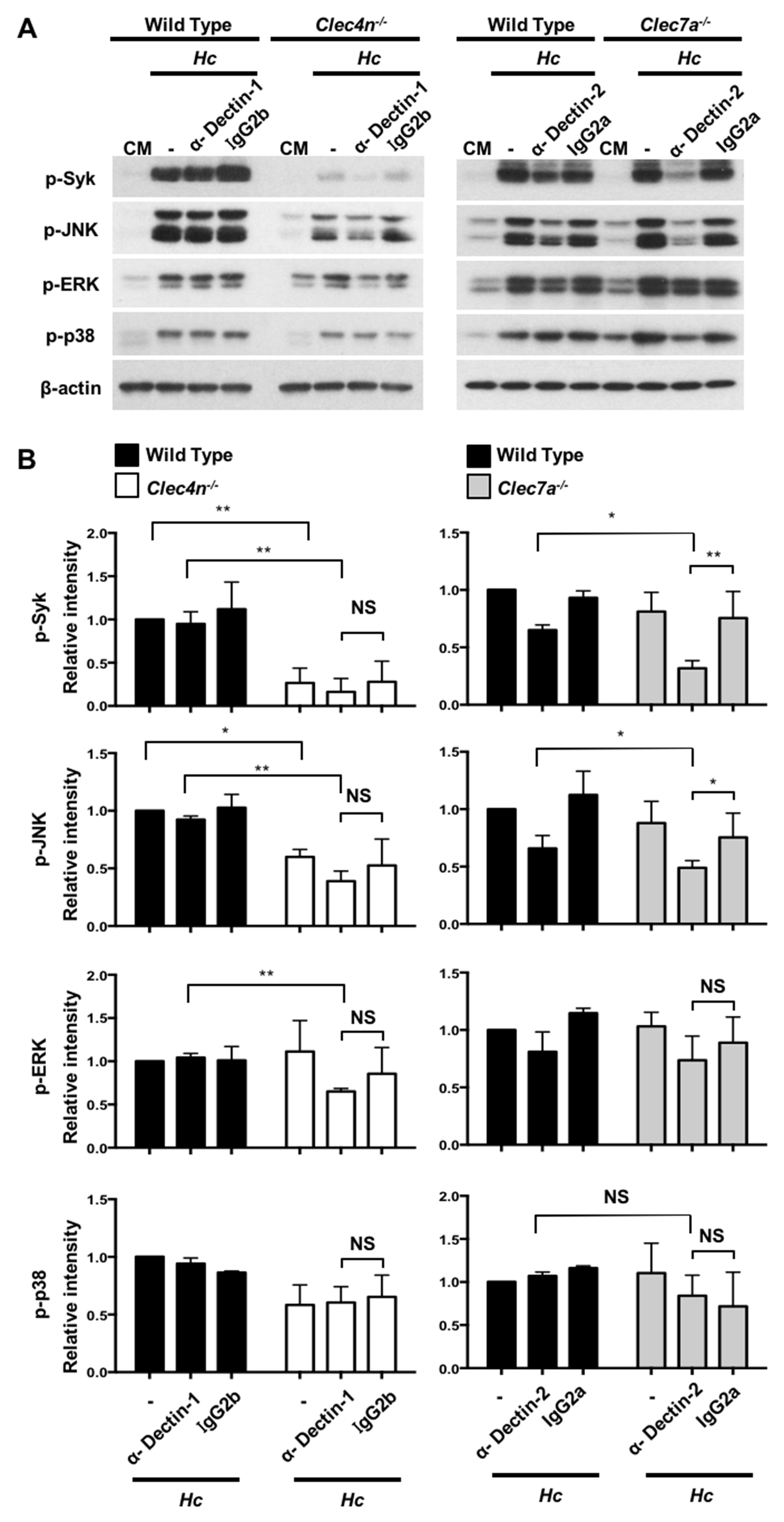

Supplement: S8 Fig — (A) BMDCs from wild type, Dectin-2-deficient (Clec4n-/-) and Dectin-1-deficient (Clec7a-/-) mice were pretreated with Dectin-1 and Dectin-2 blocking antibody, respectively, before stimulation with H. capsulatum. Collected cell lysates were analyzed for MAPK signaling molecules by Western blotting. One representative of three independent experiments is presented. (B) Relative intensity of phosphorylated MAPK molecules were quantified by ImageJ (n = 3). IgG2a and IgG2b were used as isotype controls. Error bars indicate standard deviation of the mean. * p < 0.05, ** p < 0.01, *** p < 0.001, NS, not significant [two-way ANOVA with Tukey post-hoc analysis and 2-tailed t-test (B)]. (TIF) [file ppat.1006485.s008.tif]

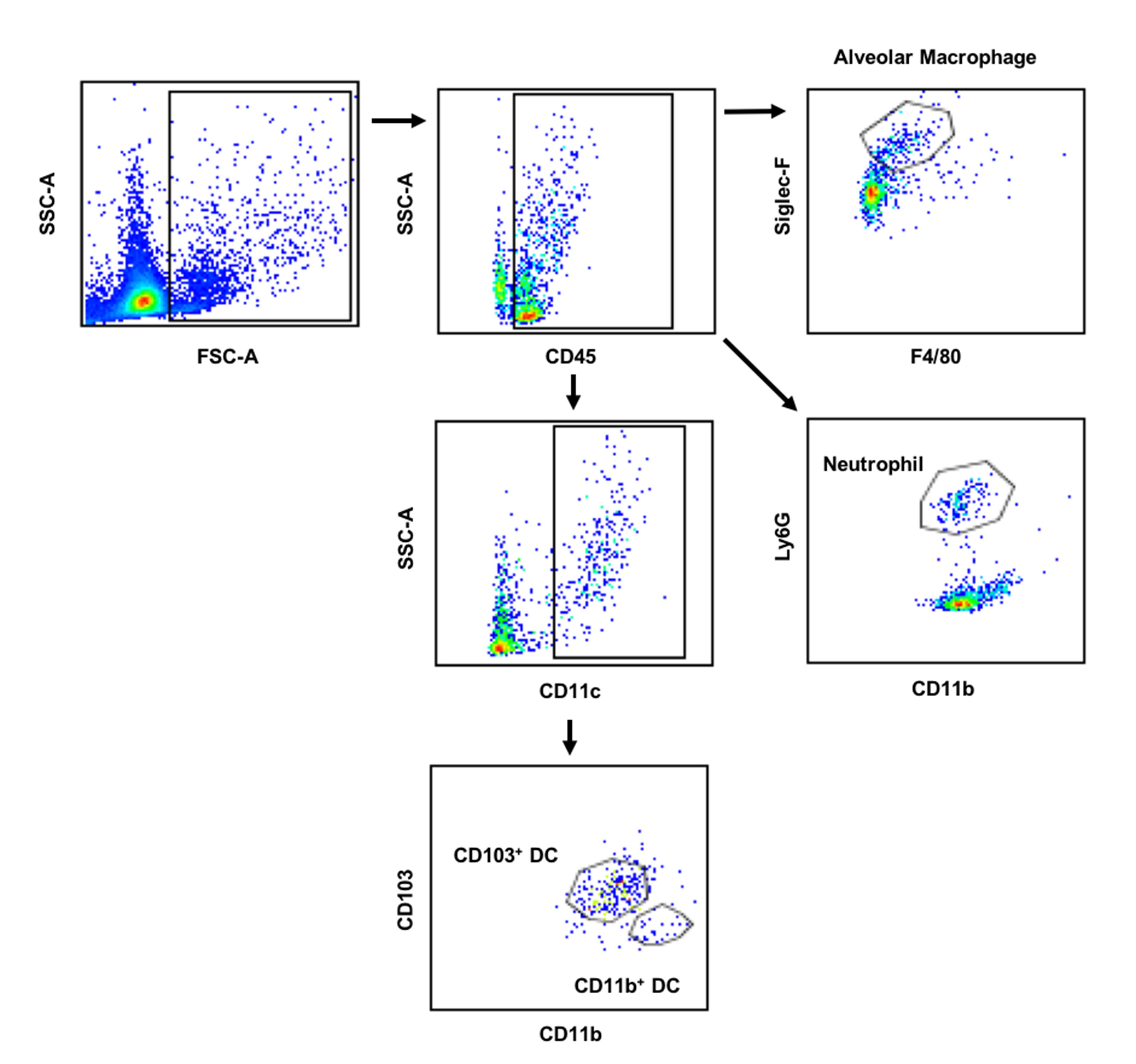

Supplement: S9 Fig — The lung cell population was determined by flow cytometry based on the following gating strategy: Viable cells were selected by gating out debris. CD45+CD11c+CD103+CD11b- cells are designated as CD103+ DC and CD45+CD11c+CD103-CD11b+ cells as CD11b+ DC, Siglec-F+F4/80+ cells as alveolar macrophages, and CD11b+Ly6G+ as neutrophils. (TIF) [file ppat.1006485.s009.tif]

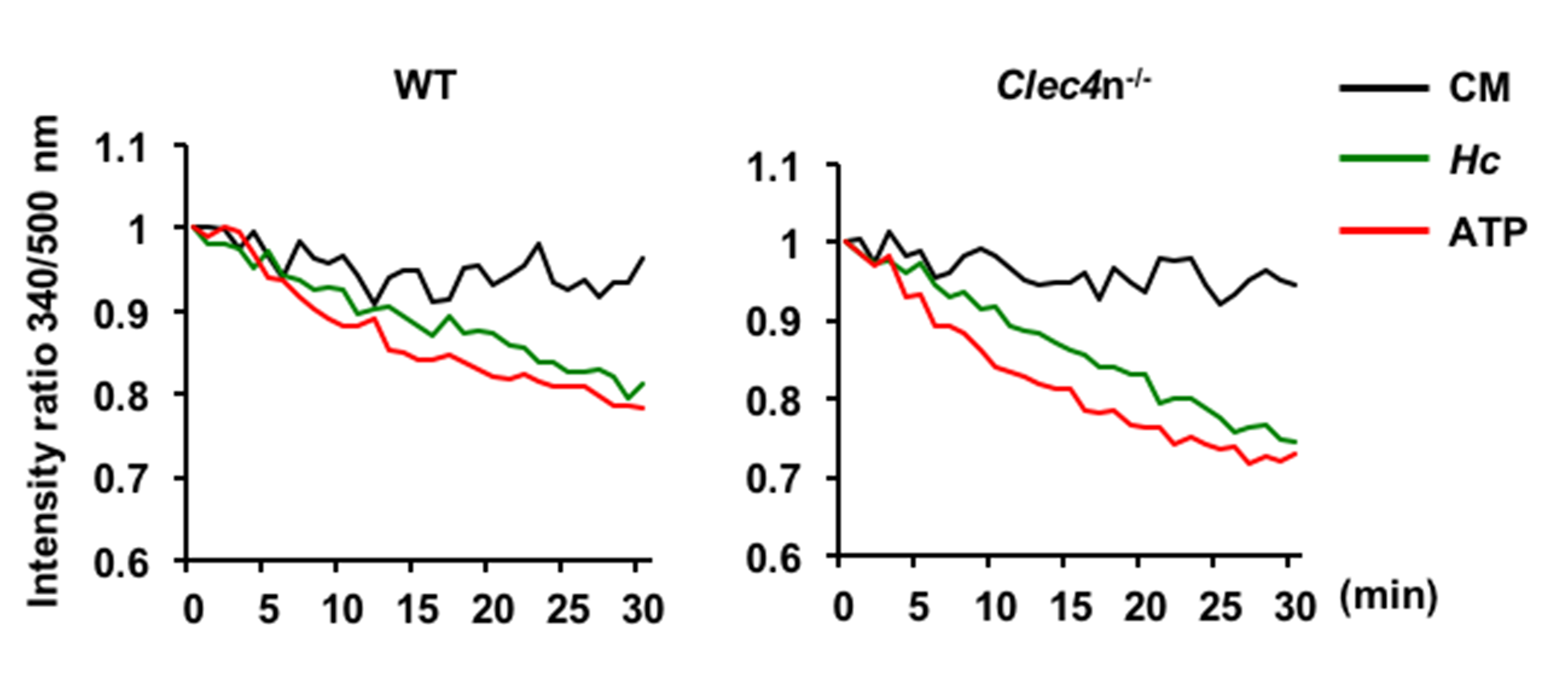

Supplement: S10 Fig — BMDCs (2 × 10^5^) from wild type and Clec4n-/- mice were incubated with potassium-sensitive probe PBFI/AM (2 μM) in the presence of Pluronic F-127 (0.05%) at room temperature in the dark for 60 min. After one wash, cells were stimulated with H. capsulatum at MOI of 1. Cells stimulation with ATP at 5 mM was used as a positive control for induction of K+ efflux. Fluorescence intensity ratio of PBFI (excitation wavelength 340 nm, emission wavelength 500 nm) was recorded every min for 30 mins. One representative of two independent experiments is presented. (TIF) [file ppat.1006485.s010.tif]

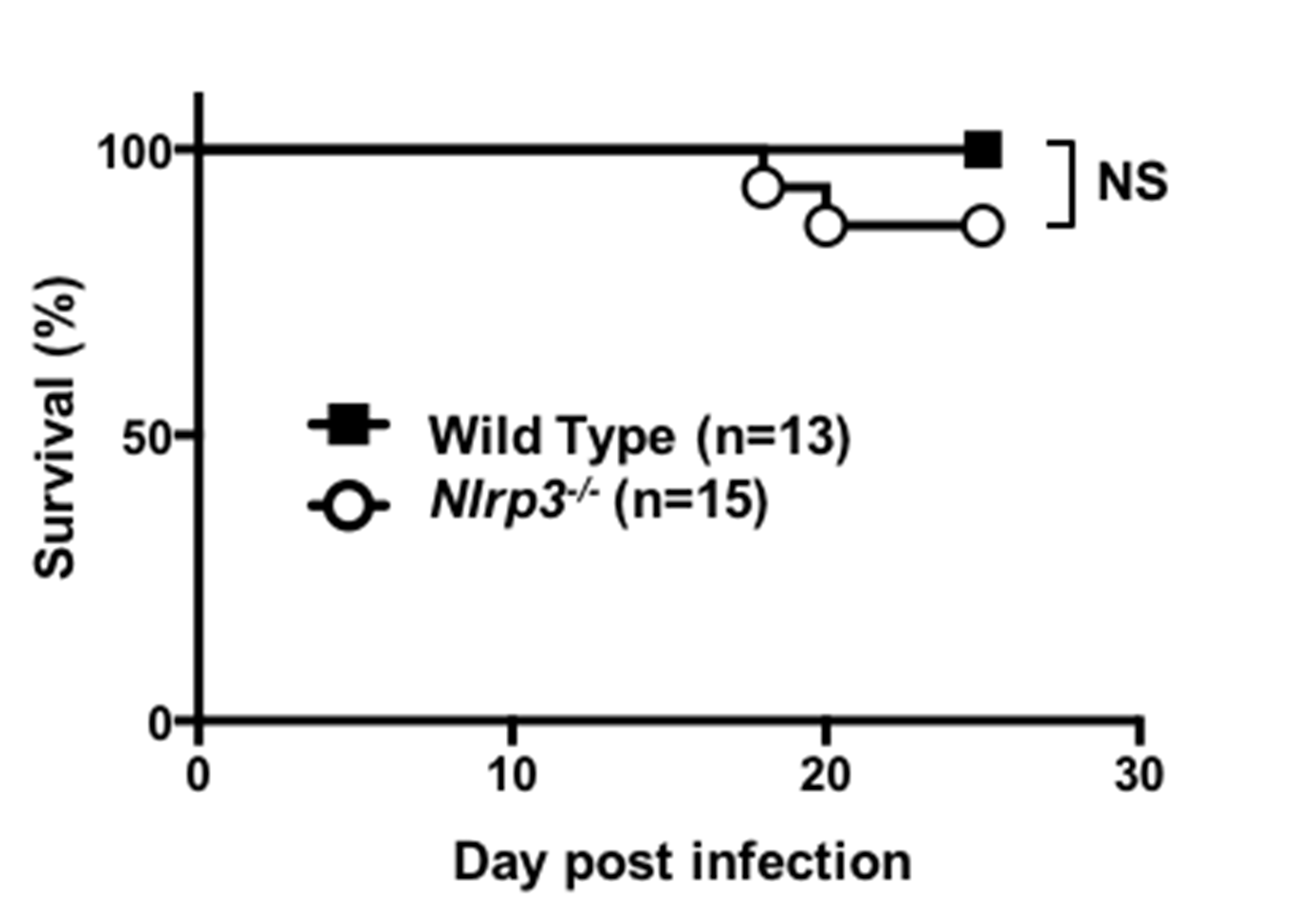

Supplement: S11 Fig — WT and Nlrp3-/- mice were intravenously infected with H. capsulatum (2 × 10^6^). Survival was analyzed by log-rank test. (TIF) [file ppat.1006485.s011.tif]

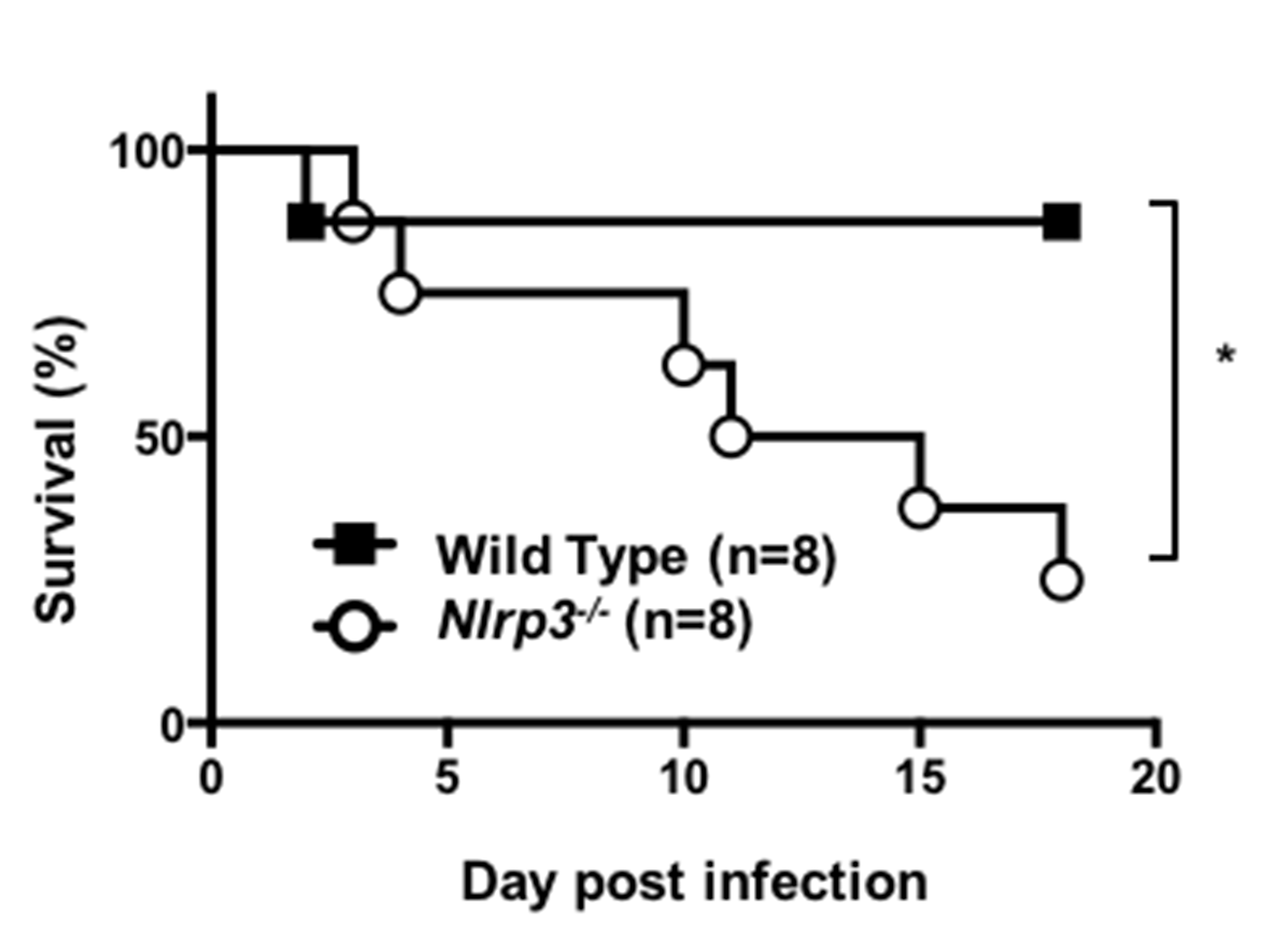

Supplement: S12 Fig — WT and Nlrp3-/- mice were intratracheally infected with H. capsulatum (1 × 10^7^). Survival was analyzed by log-rank test. * p < 0.05. (TIF) [file ppat.1006485.s012.tif]

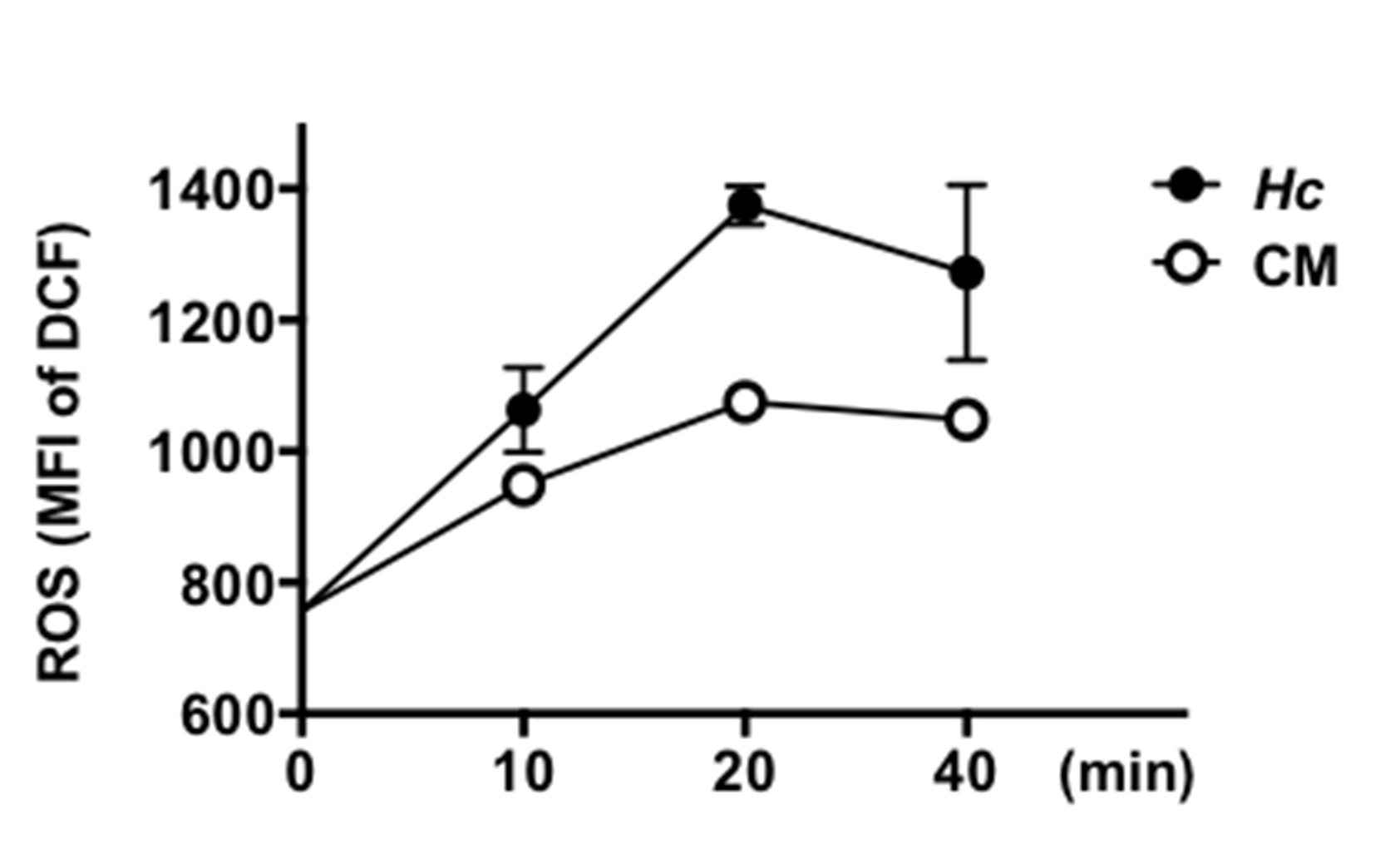

Supplement: S13 Fig — BMDCs (1.2 × 10^6^) from wild type mice were incubated with 10 μM of CM-H2DCFDA for 30 min before stimulation with or without H. capsulatum. The levels of ROS production are shown as mean florescence intensity (MFI) of oxidized DCF fluorescence (n = 3). The MFI at zero minute represents value of the control without stimulation. One representative of two independent experiments is shown. (TIF) [file ppat.1006485.s013.tif]
